# Predicting COVID-19 prognosis in hospitalized patients based on early status

**DOI:** 10.1128/mbio.01508-23

**Published:** 2023-09-08

**Authors:** David Natanov, Byron Avihai, Erin McDonnell, Eileen Lee, Brennan Cook, Nicole Altomare, Tomohiro Ko, Angelo Chaia, Carolayn Munoz, Samantha Ouellette, Suraj Nyalakonda, Vanessa Cederbaum, Payal D. Parikh, Martin J. Blaser

**Affiliations:** 1 Rutgers Robert Wood Johnson Medical School, Piscataway, New Jersey, USA; 2 Department of Medicine, Robert Wood Johnson Medical School, New Brunswick, New Jersey, USA; 3 Center for Advanced Biotechnology and Medicine, Rutgers University, New Brunswick, New Jersey, USA; Columbia University Medical College, New York, New York, USA

**Keywords:** SARS-CoV-2 infection, viral infections, machine learning, hospital medicine, biomarkers, pneumonia, ICU, coagulation, coagulopathy, renal failure

## Abstract

Predicting which patients are at greatest risk of severe disease from COVID-19 has the potential to improve patient outcomes and improve resource allocation. We developed machine learning models for predicting COVID-19 prognosis from a retrospective chart review of 969 hospitalized COVID-19 patients at Robert Wood Johnson University Hospital during the first pandemic wave in the United States, focusing on 77 variables from patients’ first day of hospital admission. Our best 77-variable model was better able to predict mortality (receiver operating characteristic area under the curve [ROC AUC] = 0.808) than CURB-65, a commonly used clinical prediction rule for pneumonia severity (ROC AUC = 0.722). After identifying highly predictive variables in our full models using Shapley additive explanations values, we generated two models, platelet count, lactate, age, blood urea nitrogen, aspartate aminotransferase, and C-reactive protein (PLABAC) and platelet count, red blood cell distribution width, age, blood urea nitrogen, lactate, and eosinophil count (PRABLE), that use age and five common laboratory tests to predict mortality (PLABAC: ROC AUC = 0.796, PRABLE: ROC AUC = 0.793), which also outperformed CURB-65. We externally validated PLABAC using data from the National COVID Cohort Collaborative Data Enclave from 7901 hospitalized COVID-19 patients from the pre-vaccination period and 1547 from the vaccination period, yielding ROC AUCs of 0.755 and 0.766, respectively. This study demonstrates that our models can accurately predict COVID-19 outcomes from a small number of variables obtained early in a patient’s hospital stay in patients from institutions around the United States after the initial pandemic wave. These models can serve as a clinical prediction aid and accurately capture a patient’s prognosis using a small number of routinely obtained laboratory values.

**IMPORTANCE:**

COVID-19 remains the fourth leading cause of death in the United States. Predicting COVID-19 patient prognosis is essential to help efficiently allocate resources, including ventilators and intensive care unit beds, particularly when hospital systems are strained. Our PLABAC and PRABLE models are unique because they accurately assess a COVID-19 patient’s risk of death from only age and five commonly ordered laboratory tests. This simple design is important because it allows these models to be used by clinicians to rapidly assess a patient’s risk of decompensation and serve as a real-time aid when discussing difficult, life-altering decisions for patients. Our models have also shown generalizability to external populations across the United States. In short, these models are practical, efficient tools to assess and communicate COVID-19 prognosis.

## INTRODUCTION

COVID-19 is an emergent health challenge with over 700 million cases around the world resulting in over six million deaths (1). Although many people infected with SARS-CoV-2 have few or no symptoms, others develop more severe complications, including acute respiratory distress syndrome and multiorgan failure (2). The ability to predict progression to severe illness has been the subject of intense study, especially as effective interventions have often been limited during outbreak scenarios, resulting in suboptimal resource allocation (3 – 5). Conversely, not allocating sufficient resources to patients at risk of serious illness may lead to unnecessary loss of life, while giving aggressive therapies to patients at low risk of dying can lead to unnecessary complications (6 – 8).

Particular demographic variables, comorbidities, and laboratory findings have been found to be significant risk factors for severe COVID-19, sometimes in ways that may have not been considered *a priori* (9 – 13). Since many front-line clinicians continue to manage numerous COVID-19 patients daily, quickly understanding the risk landscape of their patients is useful. Predictive algorithms based on machine learning techniques can assist clinicians in identifying which patients are most likely to have severe COVID-19 complications, allowing for early intervention to mitigate decompensation and improved utilization of scarce resources during high-volume scenarios (14). We now use data from a retrospective analysis collected at the start of the pandemic in the United States to build algorithms to improve prediction of COVID-19 outcomes and identify the key factors that predict the disease’s outcome using SHapley Additive exPlanations (SHAP) values (Fig. S1 and “Feature importance and generation of simplified models” in Materials and Methods). We found that this approach outperformed CURB-65, a clinical prediction rule originally developed for community-acquired pneumonia used by physicians for predicting COVID-19 outcomes (15 – 19) and performed well in two independent large national cohorts during both the prevaccination and vaccination periods.

## RESULTS

### Patient characteristics

The patient cohort used to generate our models was derived from patients hospitalized at Robert Wood Johnson University Hospital (RWJUH) with COVID-19 during the initial pandemic wave between 19 March 2020 and 31 May 2020. Of the 77 variables we chose to include in our model (Table S5), the variables significantly associated with COVID-19 mortality are summarized in Table 1. Variables related to patient outcomes such as intensive care unit (ICU) admission and treatment data were excluded from Table 1 and from our models. The patient population we studied had a relatively high frequency of co-morbidities and often had deranged vital signs at baseline (Table 1), consistent with their acute illness, and a high COVID-19 mortality rate (30.8%). As expected, in univariant analyses, there were significant associations of mortality with number of comorbidities, those related to cardiovascular fitness, hypercoagulable state, dementia, and hyperlipidemia, and with low systolic and diastolic blood pressures.

**TABLE 1:** Demographics, comorbidities, vital signs, and laboratory findings used in full variable models significantly associated with COVID-19 mortality in univariate analysis*^a^* ^, *c*^

Variable	Patients who survived	Patients who died	*P* value	SMD^*b*^
	704	217		
Age, median (Q1, Q3)	60.0 (48.0, 71.0)	74.0 (64.0, 84.0)	<0.001	0.846
Ethnicity, *n* (%)				
African American	101 (14.3)	25 (11.5)	0.007	0.403
East Asian	49 (7.0)	15 (6.9)		
Hispanic	253 (35.9)	47 (21.7)		
Other ethnicity	26 (3.7)	9 (4.1)		
South Asian	41 (5.8)	10 (4.6)		
White	234 (33.2)	111 (51.2)		
H/O arrhythmia, *n* (%)	77 (10.9)	48 (22.1)	0.003	0.305
H/O coronary artery disease or MI, *n* (%)	118 (16.8)	65 (30.0)	0.002	0.316
H/O cerebrovascular disease, *n* (%)	60 (8.5)	42 (19.4)	0.001	0.317
H/O malignancy, *n* (%)	72 (10.2)	48 (22.1)	0.001	0.327
H/O hyperlipidemia, *n* (%)	244 (34.7)	105 (48.4)	0.028	0.281
H/O dementia, *n* (%)	75 (10.7)	50 (23.0)	<0.001	0.336
Systolic blood pressure, median (Q1, Q3)	132.0 (118.0, 146.0)	123.0 (108.0, 140.0)	0.001	−0.342
Diastolic blood pressure, median (Q1, Q3)	75.0 (66.0, 83.0)	70.0 (60.0, 78.0)	<0.001	−0.403
Patient responsive, *n* (%)	657 (93.3)	175 (80.6)	<0.001	0.384
Altered mental status, *n* (%)	64 (9.1)	52 (24.0)	<0.001	0.409
AST, median (Q1, Q3)	45.0 (32.0, 71.0)	56.0 (38.0, 86.0)	0.004	0.283
Albumin, mean (SD)	3.6 (0.5)	3.4 (0.5)	<0.001	−0.433
BUN, median (Q1, Q3)	15.0 (10.0, 25.0)	30.5 (19.0, 49.8)	<0.001	0.615
CO_2_, total, median (Q1, Q3)	22.4 (20.3, 24.2)	21.1 (19.1, 23.6)	0.013	−0.257
Creatinine, median (Q1, Q3)	0.9 (0.7, 1.2)	1.3 (0.9, 1.9)	<0.001	0.219
D-dimer level, median (Q1, Q3)	1,003.0 (560.5, 1,758.8)	1,272.0 (849.0, 3,962.0)	<0.001	0.327
Lactate, median (Q1, Q3)	1.5 (1.1, 2.0)	2.1 (1.5, 3.0)	<0.001	0.573
Lymphocyte count, absolute, median (Q1, Q3)	0.9 (0.6, 1.3)	0.7 (0.5, 1.1)	0.006	−0.145
MPV, median (Q1, Q3)	8.4 (7.9, 9.1)	9.0 (8.1, 9.7)	<0.001	0.416
Platelet count, median (Q1, Q3)	229.5 (176.0, 303.5)	199.0 (154.0, 275.0)	0.007	−0.310
Potassium, median (Q1, Q3)	4.0 (3.7, 4.4)	4.2 (3.8, 4.7)	0.047	0.253
RBC MCHC, median (Q1, Q3)	33.5 (32.7, 34.1)	33.0 (32.1, 33.7)	<0.001	−0.422
RDW, median (Q1, Q3)	14.1 (13.4, 15.3)	15.2 (14.0, 17.1)	<0.001	0.460
Serum protein, total, median (Q1, Q3)	7.3 (6.8, 7.7)	7.1 (6.6, 7.5)	0.038	−0.261
Troponin T, elevated, *n* (%)	79 (15.5)	64 (38.8)	<0.001	0.542

^*a*^ Bonferroni corrected Kruskal-Wallis H test was used for all non-normal continuous variables. Bonferroni corrected two-sample *t*-test was used for albumin, the only normally distributed continuous variable. Chi-squared tests were used for all categorical variables.

^*b*^ SMD was used for assessment of effect size, with positive values indicating direct relationships and negative values indicating inverse relationships.

^*c*^ AST, alanine aminotransferase; BUN, blood urea nitrogen; H/O, history of; MCHC, mean corpuscular hemoglobin concentration; MI, myocardial infarction; MPV, mean platelet volume; Q1, first quartile; Q3, third quartile; RBC, red blood cell; RDW, red blood cell distribution width; SD, standard deviation; SMD, standardized mean difference.

### Predictive ability of full mortality models

Since our major aim was to develop a predictive model for mortality, we compared five models with CURB-65 (Table 2), using the validated cutoff value of 2 for the CURB-65 metric based on sensitivity analysis (Table S4) (15). The best-performing algorithm for predicting mortality by mean cross-validated receiver operating characterisstic area under the curve (ROC AUC) was the voting classifier, but the result for extreme gradient boosted trees (XGBoost) was similar and had a slightly improved F1 score, which is relevant in predicting patients with high likelihood of dying (Table 2). The ROC AUC was much lower for CURB-65 than for our highest-performing algorithms, but with comparable F1 score. All five algorithms had improved probability estimates for COVID-19 mortality than CURB-65, as measured by negative log loss. The voting classifier outperformed CURB-65 by providing both better mortality risk prediction, mostly driven by superior identification of low-risk patients and proper probability calibration.

**TABLE 2:** Comparison of CURB-65 with other models for mortality prediction*^a^* ^,^ *^b^*

Algorithm	No. of features	Accuracy	Sensitivity	Specificity	PPV	NPV	F1	ROC AUC	Negative log loss
**CURB-65**	**5**	**0.705**	**0.756**	**0.689**	**0.428**	**0.901**	**0.547**	**0.722**	−**0.602**
Full logistic regression	77	0.690	0.742	0.675	0.414	0.896	0.530	0.792	−0.578
Full random forest	77	0.805	0.285	0.964	0.727	0.815	0.399	0.803	−0.449
Full XGBoost	77	0.750	0.682	0.771	0.487	0.888	0.564	0.802	−0.500
Full SVM	77	0.718	0.664	0.734	0.438	0.878	0.524	0.785	−0.451
**Full voting**	**77**	**0.795**	**0.521**	**0.879**	**0.579**	**0.858**	**0.540**	**0.808**	−**0.466**
PLABAC logistic regression	6	0.706	0.713	0.703	0.425	0.890	0.532	0.782	−0.568
PLABAC random forest	6	0.768	0.571	0.828	0.510	0.863	0.536	0.796	−0.479
PLABAC XGBoost	6	0.709	0.696	0.713	0.426	0.885	0.527	0.788	−0.553
PLABAC SVM	6	0.730	0.668	0.748	0.458	0.880	0.540	0.784	−0.459
**PLABAC voting**	**6**	**0.772**	**0.580**	**0.831**	**0.514**	**0.866**	**0.543**	**0.796**	−**0.491**
PRABLE logistic regression	6	0.719	0.700	0.724	0.443	0.888	0.540	0.783	−0.567
**PRABLE random forest**	**6**	**0.787**	**0.585**	**0.849**	**0.550**	**0.870**	**0.564**	**0.793**	−**0.483**
PRABLE XGBoost	6	0.721	0.686	0.731	0.445	0.885	0.536	0.788	−0.538
PRABLE SVM	6	0.749	0.663	0.775	0.481	0.883	0.555	0.783	−0.453
PRABLE voting	6	0.769	0.580	0.827	0.512	0.866	0.539	0.792	−0.485

^*a*^ All metrics (other than CURB-65) were derived via stratified 10-fold cross-validation on our data set and averaged across the 10-folds by the mean. Top-performing models of each class are bolded.

^*b*^ NPV, negative predictive value; PPV, positive predictive value; SVM, support vector machine.

### Feature importance in mortality determinations

**FIG 1:**
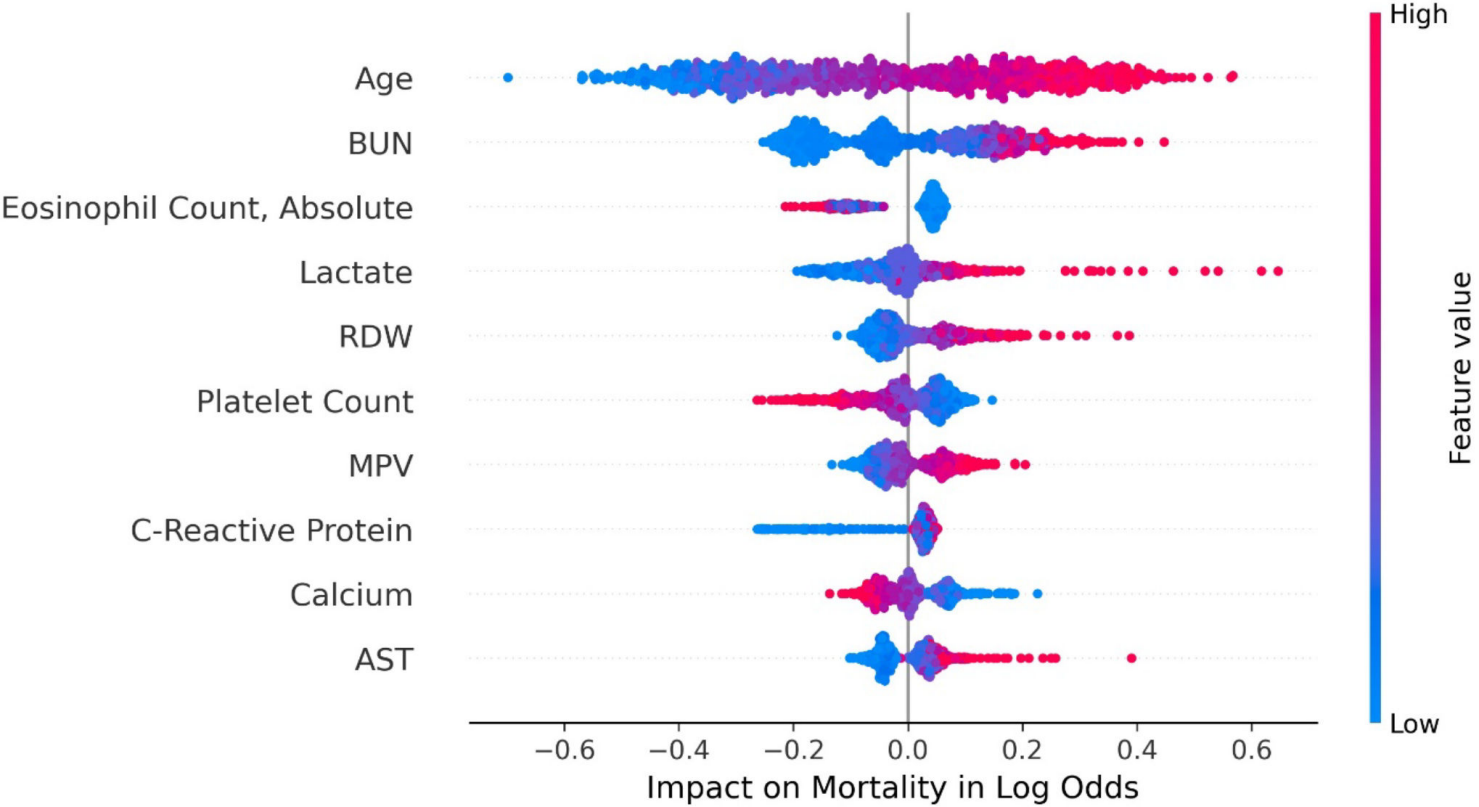
The 10 most important features identified by SHAP values for predicting mortality by the 77-variable voting classifier. Each point on the plot is a patient’s value for the specified variable, in descending ranked absolute feature importance for the voting classifier for mortality prediction. The numerical feature values are shown on a red (high)-blue (low) scale. Impact on model output is shown as log odds for mortality. Abbreviations: AST, aspartate aminotransferase; BUN, blood urea nitrogen; MPV, mean platelet volume; RDW, red blood cell distribution width.

SHAP values permit examining how single variables and combinations influence a model’s prediction of risk. Using the mortality voting classifier, the 10 most important variables in the mortality-predicting model (Fig. 1) include important components of CURB-65 with age as the strongest risk factor. We generated a six-variable model called platelet count, lactate, age, blood urea nitrogen, aspartate aminotransferase, and C-reactive protein (PLABAC), which uses variables that are both top 10 performers by SHAP values in our mortality voting classifier and are also available as part of the National COVID Cohort Collaborative Data Enclave (N3C) data set we aimed to use for external validation. We also generated an additional model called PRABLE (platelet count, red cell distribution width, age, blood urea nitrogen, lactate, and eosinophil count) that utilized the top six variables from Fig. 1. These each had similar performance to the models generated with the full set of features and outperformed the ROC AUC score generated by CURB-65 for our population (Table 1). In particular, the PLABAC voting classifier and the PRABLE random forest model were the strongest in terms of ROC AUC. We developed a web tool that enables physicians to estimate their hospitalized patient’s risk using the PLABAC voting classifier (https://plabac-bc6be52803c8.herokuapp.com/). These analyses show that age and either of two sets of five commonly obtained laboratory tests can be used for highly predictive models of mortality.

### External validation of PLABAC model on N3C data set

We validated the performance of the PLABAC model in predicting COVID-19 mortality on an external data set utilizing the N3C database, dividing the test data into data before and after 1 March 2021, to evaluate the models’ performances on patients before and during the era of mass vaccination for COVID-19 (see Table 4); two of the variables in the PRABLE model were not available in the N3C data set. We found that the PLABAC voting classifier was the strongest performer by ROC AUC in both our own data and in both external data sets (Tables 1 and 4). The model performed slightly worse overall on the two external data sets than of the population on which it was trained but overall still had strong performances on both data sets. PLABAC had a slightly higher ROC AUC and a slightly lower F1 in predicting COVID-19 prognosis in the more recent (vaccine-era) patients, which may be due to the lower COVID-19 mortality in that time frame.

### Decrease in full model performance for prediction of ICU admission and intubation

**FIG 2:**
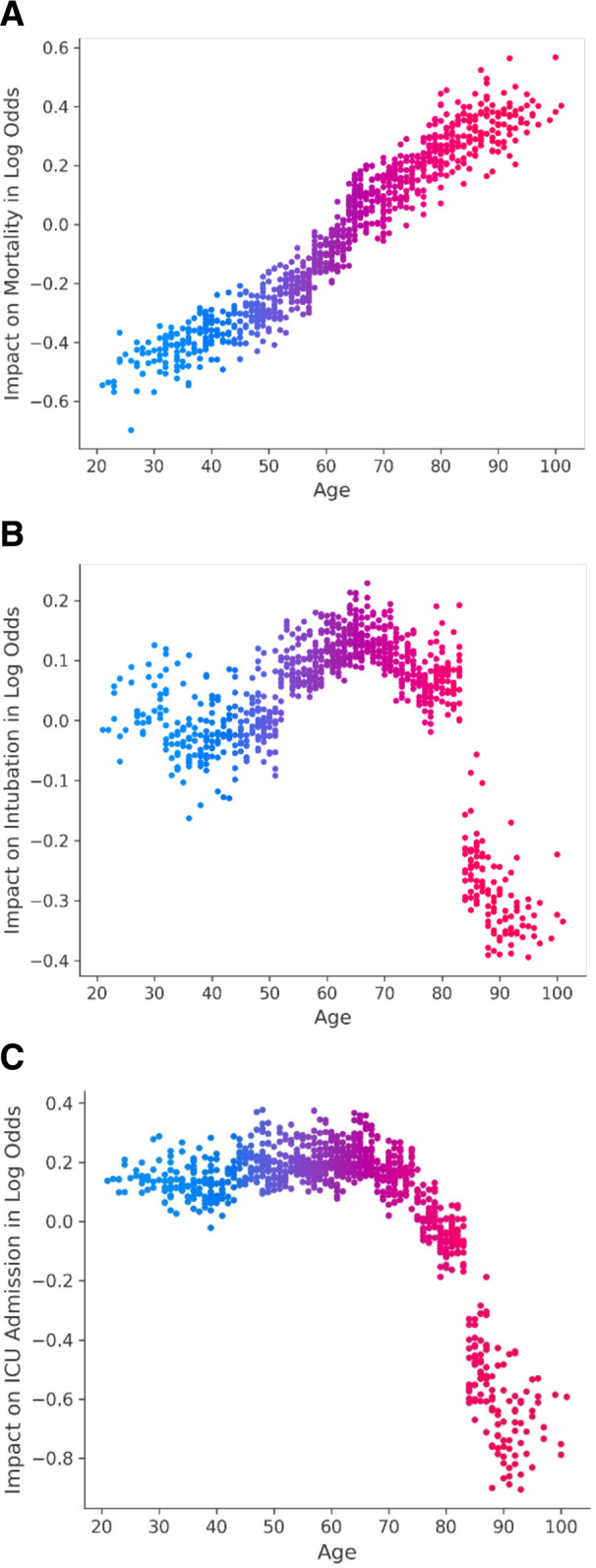
Importance of patient age in the prediction of mortality, intubation, and ICU admission. SHAP values for age. The *y* axis represents the impact of a value on our model output expressed in log odds, where a more positive number is associated with higher risk, and the *x* axis is the numerical value of age, in years.

For predicting intubation (Table 3), we considered our voting classifier the best model for analysis because it had the best ROC AUC, but the support vector machine (SVM) classifier had a slightly inferior ROC AUC with a better F1 score. The XGBoost algorithm was our strongest for predicting ICU admission by ROC AUC, but the voting classifier had a far better F1 score (Table 3). However, all these models were not as accurate in predicting intubation and ICU admission compared to predicting mortality. The calculated SHAP values for the features weighed in predicting ICU admission and intubation are instructive (Fig. S2 and S3). Many of the requisite variables have been well validated as individual risk factors, are consistent with proposed COVID-19 disease mechanisms, and are predictive of mortality in our model and in other models (15 – 18, 20 – 24). However, other risk factors for intubation and ICU admission in our voting classifier model were more complex. Advanced age was associated with lower likelihood of intubation and ICU admission, despite their clear link to higher mortality likelihood (Fig. 2). Similarly, a history of dementia predicted a lower chance of ICU admission or intubation, but increased odds of death (Fig. S4). These conflicting observations may indicate that our models for these two outcomes captured the allocation of scarce resources (triaging) during the initial COVID-19 wave rather than a true reflection of patient necessity for intensive treatment.

**TABLE 3:** Comparison of full-feature algorithms for intubation and ICU admission^*a*^

Algorithm	No. of features	Accuracy	Sensitivity	Specificity	PPV	NPV	F1	ROC AUC	Negative log loss
Intubation lgistic regression	77	0.666	0.643	0.671	0.313	0.891	0.420	0.729	−0.622
Intubation random forest	77	0.811	0.023	0.995	0.333	0.814	0.043	0.761	−0.430
Intubation XGBoost	77	0.830	0.293	0.955	0.621	0.853	0.391	0.754	−0.464
Intubation SVM	77	0.737	0.585	0.772	0.377	0.889	0.457	0.752	−0.426
**Intubation voting**	**77**	**0.826**	**0.242**	**0.963**	**0.612**	**0.845**	**0.343**	**0.767**	−**0.426**
ICU logistic regression	77	0.705	0.756	0.689	0.428	0.901	0.547	0.722	−0.602
ICU random forest	77	0.680	0.610	0.699	0.362	0.867	0.452	0.711	−0.615
**ICU XGBoost**	**77**	**0.796**	**0.165**	**0.971**	**0.613**	**0.808**	**0.249**	**0.747**	−**0.480**
ICU SVM	77	0.782	0.390	0.890	0.494	0.841	0.430	0.731	−0.482
ICU voting	77	0.732	0.540	0.785	0.416	0.861	0.467	0.735	−0.463

^*a*^ All metrics were derived via stratified 10-fold cross-validation on our data set and averaged across the 10-folds by the mean. Intubation and ICU models were evaluated to predict those outcomes, respectively. Top-performing models of each class are bolded.

## DISCUSSION

Overall, we developed two algorithms that use a small number of variables that outperformed CURB-65 in predicting COVID-19 mortality, one of which was shown to have external validity. Although our algorithms performed similarly to CURB-65 in identifying patients at high mortality risk, in general, they were far better in identifying low-risk patients, therefore improving reliability. Our ability to predict intubation and ICU admission was weaker than mortality prediction, which may reflect clinical decisions related to triage during an overwhelming epidemic wave (11, 25, 26).

Our best classifiers for mortality prediction by ROC AUC performed better or similarly than in some studies (10, 11, 27 – 30) but worse than in others (10, 31 – 39). However, the optimal way to compare these predictive models is to assess their generalizability to other populations. Variation in patient population, sample size, use of validation sets vs stratified cross-validation, hospital length of stay (LOS), and patient selection all affect algorithm performance. This is highlighted by the poor performance of CURB-65 in our population compared to other studies (15 – 18). While CURB-65 is reasonably sensitive for COVID-19 prognosis, it is limited by its lack of specificity, which reduces its applicability to the real world of clinical medicine. Comparisons with prior COVID-19 prediction model outcomes are limited by differences in populations, disease severity, treatment modalities available, and methodologies.

Although there have been prior risk scores for COVID-19 prognosis (10, 31 – 39), we believe ours is an improvement for several reasons. First, our risk models, PLABAC and PRABLE, include only a small number of high-impact variables, which to our knowledge is unique among similar works. Our algorithms, trained on a diverse US-based population, may be particularly useful for US clinicians, which is further supported by the generalizability of the PLABAC model. Finally, we validated our model, unlike in prior studies, showing that even for patients in 2021, past the worst days of triaging and ventilator shortages in the United States (40), the PLABAC model performed well in prognosticating across a wide range of hospital environments; this provides confidence that the model’s predictions are generalizable.

A second strength is that using an algorithm based on only six routinely gathered variables to predict mortality may provide utility for frontline clinicians. Aggregating a patient’s risk of death into a six-variable package is especially useful in circumstances in which a clinician quickly needs to make rapid decisions. The models we have constructed are easy to adopt and are not only of theoretical interest but also practical to enable difficult, life-altering clinical decisions. Early identification of high-risk patients may enable physicians to monitor more closely, begin treatments earlier, and facilitate discussions about prognosis with patients and their family. Such scores can also be used to communicate quickly to other providers about prognosis and can be used in a manner similar to other prognostic scores such as the model for end-stage liver disease or the CHADS score used for stroke prognostication (41, 42). Importantly, our models are often able to predict a patient’s course months before demise or hospital discharge. Perhaps more valuable to a clinician is not only the binary variable of mortality but also the actual probability of demise or not, which provides a sense of the model’s confidence. We have used the 50% chance of mortality as the cut-off, but this treats a 90% and 51% chance of predicted mortality in exactly the same way. In this respect, the PLABAC model represents a significant improvement over CURB-65. The combination of a small number of variables and external validation aligns our models with the intrinsic pathophysiology of COVID-19 and not a temporal or institutional quirk. With novel vaccine-resistant COVID-19 variants appearing regularly and COVID-19 remaining the fourth leading cause of overall mortality in the United States (43), the need for a rapid method to assess COVID-19 outcome remains high (19).

Our predictive performances may be conservative compared to others since (i) we intentionally excluded patients hospitalized for <24 h, either due to death in that time frame or mild illness and discharge to home, because this represents an obvious dichotomy to clinicians; (ii) our time frame for mortality events was longer than in other studies (27 – 29), including patients who had protracted hospital courses before their demise; (iii) we limited analysis to variables likely available to physicians within the first 24 h of hospitalization, which excludes such factors as number of ICU admissions and later therapeutic interventions (30, 37); (iv) our models were designed to sacrifice specificity and overall ROC AUC to favor better identification of illness severity, which was less common but more important to recognize; and (v) several prior COVID-19 outcome predictive algorithms used radiographic biomarkers, which may not be readily available or consistently interpreted under epidemic conditions (32, 35, 37) and were not used in our study.

This study has several important limitations; it was conducted from a data set from a single institution in early 2020 at the start of the COVID-19 pandemic in the United States; models generated from these data may not be fully representative of COVID-19 patients throughout the United States. Differing triage thresholds also may limit generalizability of algorithms predicting intubation and ICU admission since they depend on local epidemic status. The counter-intuitive differences in the predictive values of age and dementia for mortality on the one hand and ICU admission/intubation on the other may reflect such physician triage, explaining the reduced algorithm accuracy predicting those events (25, 26) Because we were unable to obtain symptom data in our external 2021 validation data set, we were unable to assess how CURB-65 performed on the external data set.

It also is important to consider that the selection of the patients included in the training and testing of the model affects its performance and use cases. Our models are designed to be used as general-purpose tools to stratify the mortality risk for all hospitalized COVID-19 patients within the critical first 24 h of their hospital stay, which can aid in triaging patients before there is obvious clinical evidence of decompensation. This approach enables outcome prediction during the most uncertain portion of a patient’s hospital stay. This approach enables outcome prediction during the portion of a patient’s hospital stay with the greatest prognostic uncertainty. While we attempted to tailor our models to this end by excluding patients hospitalized for less than 24 h, some of the predictive performance of the model will be affected by patients who are already decompensated upon arrival to the hospital or conversely represent hospital admissions with a low likelihood of serious illness. Future directions of our study include focusing on COVID-19 patients in the ICU and generating a rolling estimate of prognosis by continually incorporating new clinical information. An important limitation of observational studies such as we present is the confounding that is inherent to retrospective studies. We hope to address this limitation and to refine our model’s performance using a prospective cohort of COVID-19 patients in the future.

Our study used SHAP values to identify important factors in predicting severe COVID-19 outcomes; however, most comorbidities and demographic variables were not highly predictive. Advanced age, a well-documented risk factor for severe COVID-19 (2, 9 – 12, 15, 20, 41, 42), was the strongest predictor of mortality in our study and most likely accounted for the highest mortality rates in non-Hispanic whites, who were older on average in our studied population. Abnormal blood urea nitrogen (BUN) also is a well-validated prognostic marker of severity (15, 23), as COVID-19 causes renal injury through hypotension, direct podocyte infection, and immune dysregulation following cytokine storm (23, 44, 45). Our models highlighted elevated lactate, reflecting both abnormal metabolism and hypoxia, previously implicated in poor COVID-19 prognosis (9, 18, 46). Low platelet count is an indicator of thrombotic activity and platelet turnover, which predispose to the severe COVID-19 complications of stroke and disseminated intravascular coagulation (13, 47, 48). Red blood cell distribution width (RDW), a marker of variability of red blood cell size, has been linked to adverse COVID-19 outcomes through its association with inflammation, hemolysis, and intravascular coagulopathy (49 – 51). Low eosinophil count also was a useful predictor of poor COVID-19 outcomes, consistent with prior reports documenting its perturbation (20, 21, 24, 52 – 54). Since eosinophil count was not identified in univariate analysis (Table 1), its relationship with COVID-19 may not be linear. We considered whether eosinophil count could be confounded by corticosteroid administration, which can lower eosinophil count and may hold a mortality benefit for COVID-19. Because only 66 (7.2%) of the 921 patients in our cohort were known to have been administered steroids while 421 members of the cohort had an absolute eosinophil count of 0, we believe that the effect was unaffected by the variable exposure. This is further supported by the body of evidence linking COVID-19 outcomes with eosinophil count, including one study that excluded patients given corticosteroids (55). Aspartate aminotransferase (AST) and C-reactive protein (CRP) are markers of liver damage and inflammation, and both are linked with poor COVID-19 prognosis (13, 48, 56 – 58). Overall, the risk factors identified are consistent with an illness leading to mortality due to coagulopathy, inflammation, and decreased oxygenation of critical organs. The variables that are most predictive in our models highlight that COVID-19 is a multisystemic disease with significant morbidity and mortality occurring because of coagulopathy and direct damage to critical organs such as the lung, kidney, heart, and liver. Such pathophysiology separates COVID-19 from other infections that are essentially respiratory such as influenza and bacterial pneumonia. This difference may explain why the CURB-65 score, as well as other clinical prediction rules, may not suffice for COVID-19 prognosis.

Despite the limitations above, that PLABAC still performed relatively well on the broader external data set is a sign of its utility and promise. We envision PLABAC and PRABLE being used by clinicians to predict prognosis of their patients with COVID-19, efficiently allocate resources, and facilitate communication about an individual patient’s chances of survival. Overall, this work has generated models that can create clinically useful predictions for patients being hospitalized for COVID-19 and represents a practical first step for the inclusion of machine learning into clinical decision-making for COVID-19 that can serve as a template for future prognostic models.

## MATERIALS AND METHODS

### Data collection and patient population

We performed a retrospective analysis of 969 adults who were admitted between 19 March 2020 and 31 May 2020 to the RWJUH in New Brunswick with a diagnosis of COVID-19 infection during the peak of the first wave in New Jersey. Through a retrospective chart review, the authors systematically collected data including demographics, comorbidities, symptoms, and inpatient labs, vitals, and medical management. The data were then cleaned over multiple courses of quality control, with each chart reviewed by ≥2 independent readers. Comorbidities, reported from the chart to remove surveyor bias, were later reclassified by relevant category as validated by two independent observers (Tables S1 and S2), and all laboratory values were downloaded from the hospital database. All 935 patients who tested positive for SARS-CoV-2 infection by nasopharyngeal swab using PCR were included in our study, except for 16 patients who had a hospital stay of <24 h. Patients who were diagnosed with COVID-19 clinically but were never PCR-confirmed were also excluded from the model (*n* = 34). Patients readmitted to the hospital including after the pre-specified end date were included. Time 0 was defined as the first available time stamp of a patient’s initial laboratory value obtained from either the hospital or the emergency room. Additional data included length of stay, readmission number, number of readmissions, discharge location (rehabilitation facility, home, skilled nursing facility, or death), ICU LOS, and date of death.

### Outcomes

We developed models to predict three clinical outcomes indicative of illness severity: intensive care unit admission, intubation, or death. Intubation was defined as any form of mechanical ventilation. Death was defined as a patient dying in the hospital or being discharged to hospice care, as detailed in Table S3.

### Feature processing and selection

All models began with all variables recorded within the first day of hospital admission. We assumed that binary patient history terms such as pregnancy, smoking history, comorbidities, physical examination findings, and symptoms not recorded in a patient’s chart were not present. The models included only categorical features that were present in >5% of our patient population, which excludes some of the comorbidities listed in Table S1. Ethnicity and sex, used in their constitutive categories, were treated as binary variables. Continuous variables with values in ≥50% of our patient population were included, and values that were obvious recording errors were removed. We imputed the results of missing continuous variables using the median value in our patient population. To reduce selection bias, we assessed whether the proportion of missing laboratory tests differed between patients who had a severe illness (ICU admission, intubation, or death) or not and excluded those tests whose values were not missing at random (independent *t*-test, *P* < 0.001). Tests quantifying urine blood or leukocyte esterase were converted to an ordinal scale. We calculated variance inflation factors (VIFs) for our data set to identify correlated variables, removing those with VIF of >5 from analyses (59). Some variables that are often used in concert clinically, such as history of chronic kidney disease, and serum levels of BUN and creatinine were included in our analyses despite their representing a similar underlying pathology. Our rationale was that the interaction between these variables may hold prognostic utility, which mirrors how they are used in clinical practice. We subsequently identified that patients who were unresponsive or had altered mental status were significantly (*P* < 0.05, independent *t*-test for each) less likely to have recorded symptoms, likely because such patients cannot provide a reliable history; we thus removed these symptoms from our analysis. Following this step, each variable was standardized by removing the mean and scaling to unit variance. These steps yielded a set of 77 variables relating to demographics, medical history, symptoms, physical exam findings, and laboratory values obtained early in the hospital course (Table S3).

### Machine learning analysis

We initially selected four machine learning algorithms (LASSO [least absolute shrinkage and selection operator] logistic regression, random forest, extreme gradient boosted trees, and SVM) based on prior utility for similar tasks (10, 11, 31). Each model was evaluated using 10-fold stratified cross-validation on our own data set. Algorithms were evaluated for sensitivity, specificity, positive predictive value, negative predictive value, F1 metric, receiver operating characteristic area under the curve (ROC AUC), and negative log loss value, as described (10, 12, 15, 16, 27, 30, 32, 33, 36, 38, 57, 60, 61). Class weight was balanced by the ratio of minority to majority class. Hyperparameters for each algorithm were optimized using a random search through the parameter space. A voting classifier was generated using input from the four machine learning models and used soft voting, averaging the predicted probabilities generated by each model. For analysis of mortality, algorithms were compared to CURB-65, a 5-point metric (including confusion, elevated age, BUN and respiratory rate, and decreased systolic or diastolic blood pressure) for assessing community-acquired pneumonia severity (CURB-65 score), with scores corresponding to 30-day mortality risk (15). These probabilities were used to calculate the negative log loss value of CURB-65 and a score of 2 used for the mortality cutoff, as described (15) and validated on our own cohort (Table S3).

### Feature importance and generation of simplified models

Feature importance was calculated using SHapely Additive exPlanations (SHAP) (62). Shapley values use game theory to fairly assign payout in a task by measuring the contribution of each of the players in the task and their interactions and can be used in machine learning to explain how combinations of variables contribute to the final prediction in log_e_ odds (referred to herein as log odds). We used SHAP values to generate the most predictive variables for COVID-19 prognosis. Using the top 10 most predictive variables by SHAP values from our strongest 77-variable classifier (the voting classifier), we developed two six-variable models. PLABAC uses variables represented both in this top 10 list and in the National COVID Cohort Collaborative Data Enclave data set we used for external validation, and PRABLE uses the top six variables.

### External validation

To assess our PLABAC model with clinical data external to our institution and time frame, we used data from the N3C data set, which includes patients with any encounter after 1 January 2020 and before 24 September 2021. Included patients had one of a set of *a priori*-defined SARS-CoV-2 laboratory tests, a strong positive diagnostic code, or two weak positive diagnostic codes during the same encounter or same date for patients admitted prior to May 2020 (39, 63). The cohort definition is publicly available on GitHub (64). For our validation data set, we established the definition that for a patient to be included, we needed access to their age and all five day 1 laboratory values, and that they had an inpatient hospital stay of >1 day with laboratory-confirmed COVID-19. We used 1 March 2021 as a breakpoint between the pre-vaccine period and post-vaccine period of the COVID-19 pandemic until 24 September 2021. For these two periods, 7901 (with 1863 [23.6%] deaths) and 1547 (with 285 [18.4%] deaths) patient records met our criteria and were used to evaluate the performance of our models (Table 4).

**TABLE 4:** Performance of PLABAC models on N3C data between 1 January 2021 and 1 March 2021 (*n* = 7,901) and between 1 March 2021 and 24 September 2021 (*n* = 1,547)^*a*^

Population	Algorithm	Accuracy	Sensitivity	Specificity	PPV	NPV	F1	ROC AUC	Negative log loss
Pre-vaccine	Logistic regression	0.746	0.695	0.680	0.401	0.879	0.509	0.746	−0.599
	Random forest	0.725	0.578	0.771	0.438	0.856	0.498	0.742	−0.542
	XGBoost	0.660	0.727	0.640	0.384	0.884	0.502	0.746	−0.604
	SVM	0.696	0.670	0.704	0.411	0.874	0.510	0.749	−0.479
	**Voting**	**0.735**	**0.583**	**0.782**	**0.452**	**0.859**	**0.509**	**0.755**	−**0.529**
Vaccine	Logistic regression	0.760	0.695	0.679	0.328	0.908	0.446	0.760	−0.598
	Random forest	0.740	0.572	0.778	0.368	0.889	0.448	0.747	−0.522
	XGBoost	0.686	0.726	0.677	0.337	0.916	0.461	0.753	−0.598
	SVM	0.696	0.670	0.702	0.337	0.904	0.448	0.758	−0.430
	**Voting**	**0.754**	**0.586**	**0.792**	**0.388**	**0.894**	**0.467**	**0.766**	−**0.513**

^*a*^ Models trained off of PLABAC variables from our own data set and evaluated on N3C data set from before and after the widespread adoption of COVID-19 vaccination in the United States, defined as between 1 January 2021 and 1 March 2021 (*n* = 7901) and between 1 March 2021 and 24 September 2021 (*n* = 1547). Top-performing models for each population are bolded.

### Statistical analysis

For the univariate analyses listed in Table 1, continuous variable normality was assessed through the D’Agostino-Pearson test. Bonferroni-corrected Kruskal-Wallis H test was used for all non-normal continuous variables. Bonferroni-corrected two-sample *t*-test was used for albumin, the only normally distributed continuous variable. Chi-squared tests were used for all categorical variables.

### Computation

All analysis was conducted using Python v.3.9.7. LASSO logistic regression, random forest, support vector machines, and machine learning metric calculations were implemented using scikit-learn v.0.24.2 (65). XGBoost was implemented using XGBoost v.1.5.2 (66). NumPy v.1.20.1 and pandas v.1.3.4 were used for data handling (67, 68). Statistics were calculated using scipy v.1.7.1 (69) and statmodels v.0.12.2. Figures were made with Matplotlib v.1.20.3 and Seaborn v.0.11.2 (70, 71), and SHAP v.0.40.0 was used to assess feature importance (62). Table 1 was generated via Python package, tableone v.0.7.10 (72).

## SUPPLEMENTAL MATERIAL

Fig. S1 mbio.01508-23-s0001.docx Study design.

Fig. S2 mbio.01508-23-s0002.docx Feature importance identified by SHAP values for the prediction of intubation by the voting classifier.

Fig. S3 mbio.01508-23-s0003.docx Feature importance identified by SHAP values for the prediction of ICU admission by XGBoost.

Fig. S4 mbio.01508-23-s0004.docx Importance of dementia in the prediction of mortality, intubation, and ICU admission.

Table S1 mbio.01508-23-s0005.docx Binning methods for symptoms.

Table S2 mbio.01508-23-s0006.docx Binning methods for comorbidities.

Table S3 mbio.01508-23-s0007.docx Binning methods for hospital discharge.

Table S4 mbio.01508-23-s0008.docx Comparison of CURB-65 performance at six cutoff values.

Table S5 mbio.01508-23-s0009.docx Variables used in full model for mortality.

ASM does not own the copyrights to Supplemental Material that may be linked to, or accessed through, an article. The authors have granted ASM a non-exclusive, world-wide license to publish the Supplemental Material files. Please contact the corresponding author directly for reuse.

## References

[1] Anonymous . 2022. WHO COVID-19 dashboard. World Health Organization, Geneva.

[2] Wiersinga WJ , Rhodes A , Cheng AC , Peacock SJ , Prescott HC . 2020. Pathophysiology, transmission, diagnosis, and treatment of Coronavirus disease 2019 (COVID-19): a review. JAMA 324:782–793. doi:10.1001/jama.2020.12839 32648899

[3] Truog RD , Mitchell C , Daley GQ . 2020. The toughest triage—allocating ventilators in a pandemic. N Engl J Med 382:1973–1975. doi:10.1056/NEJMp2005689 32202721

[4] Kadri SS , Sun J , Lawandi A , Strich JR , Busch LM , Keller M , Babiker A , Yek C , Malik S , Krack J , Dekker JP , Spaulding AB , Ricotta E , Powers JH , Rhee C , Klompas M , Athale J , Boehmer TK , Gundlapalli AV , Bentley W , Datta SD , Danner RL , Demirkale CY , Warner S . 2021. Association between caseload surge and COVID-19 survival in 558 U.S. hospitals, March to August 2020. Ann Intern Med 174:1240–1251. doi:10.7326/M21-1213 34224257 PMC8276718

[5] Ebinger JE , Lan R , Driver M , Sun N , Botting P , Park E , Davis T , Minissian MB , Coleman B , Riggs R , Roberts P , Cheng S . 2022. Seasonal COVID-19 surge related hospital volumes and case fatality rates. BMC Infect Dis 22:178. doi:10.1186/s12879-022-07139-2 35197000 PMC8864601

[6] Griesdale DEG , Bosma TL , Kurth T , Isac G , Chittock DR . 2008. Complications of endotracheal intubation in the critically ill. Intensive Care Med 34:1835–1842. doi:10.1007/s00134-008-1205-6 18604519

[7] Gupta VK , Alkandari BM , Mohammed W , Tobar AM , Abdelmohsen MA . 2021. Ventilator associated lung injury in severe COVID-19 pneumonia patients - case reports: ventilator associated lung injury in COVID-19. Eur J Radiol Open 8:100310. doi:10.1016/j.ejro.2020.100310 33364262 PMC7750144

[8] Grennan D , Wang S . 2019. Steroid side effects. JAMA 322:282. doi:10.1001/jama.2019.8506 31310300

[9] Han J , Shi L-X , Xie Y , Zhang Y-J , Huang S-P , Li J-G , Wang H-R , Shao S-F . 2020. Analysis of factors affecting the prognosis of COVID-19 patients and viral shedding duration. Epidemiol Infect 148:e125. doi:10.1017/S0950268820001399 32580792 PMC7332754

[10] Kim H-J , Han D , Kim J-H , Kim D , Ha B , Seog W , Lee Y-K , Lim D , Hong SO , Park M-J , Heo J . 2020. An easy-to-use machine learning model to predict the prognosis of patients with COVID-19: retrospective cohort study. J Med Internet Res 22:e24225. doi:10.2196/24225 33108316 PMC7655730

[11] Heldt FS , Vizcaychipi MP , Peacock S , Cinelli M , McLachlan L , Andreotti F , Jovanović S , Dürichen R , Lipunova N , Fletcher RA , Hancock A , McCarthy A , Pointon RA , Brown A , Eaton J , Liddi R , Mackillop L , Tarassenko L , Khan RT . 2021. Early risk assessment for COVID-19 patients from emergency department data using machine learning. Sci Rep 11:4200. doi:10.1038/s41598-021-83784-y 33603086 PMC7892838

[12] Liu D , Cui P , Zeng S , Wang S , Feng X , Xu S , Li R , Gao Y , Yu R , Wang Y , Yuan Y , Li H , Jiao X , Chi J , Liu J , Yu Y , Zheng X , Song C , Jin N , Gong W , Liu X , Cai G , Li C , Gao Q . 2020. Risk factors for developing into critical COVID-19 patients in Wuhan, China: a multicenter, retrospective, cohort study. EClinicalMedicine 25:100471. doi:10.1016/j.eclinm.2020.100471 32840491 PMC7391125

[13] Pourbagheri-Sigaroodi A , Bashash D , Fateh F , Abolghasemi H . 2020. Laboratory findings in COVID-19 diagnosis and prognosis. Clin Chim Acta 510:475–482. doi:10.1016/j.cca.2020.08.019 32798514 PMC7426219

[14] Obermeyer Z , Emanuel EJ . 2016. Predicting the future—big data, machine learning, and clinical medicine. N Engl J Med 375:1216–1219. doi:10.1056/NEJMp1606181 27682033 PMC5070532

[15] Guo J , Zhou B , Zhu M , Yuan Y , Wang Q , Zhou H , Wang X , Lv T , Li S , Liu P , Yang Y , He P , Zhang P . 2020. CURB-65 may serve as a useful prognostic marker in COVID-19 patients within Wuhan, China: a retrospective cohort study. Epidemiol Infect 148:e241. doi:10.1017/S0950268820002368 32998791 PMC7573457

[16] Satici C , Demirkol MA , Sargin Altunok E , Gursoy B , Alkan M , Kamat S , Demirok B , Surmeli CD , Calik M , Cavus Z , Esatoglu SN . 2020. Performance of pneumonia severity index and CURB-65 in predicting 30-day mortality in patients with COVID-19. Int J Infect Dis 98:84–89. doi:10.1016/j.ijid.2020.06.038 32553714 PMC7293841

[17] Brabrand M , Henriksen DP . 2018. CURB-65 score is equal to NEWS for identifying mortality risk of pneumonia patients: an observational study. Lung 196:359–361. doi:10.1007/s00408-018-0105-y 29541854

[18] Frenzen FS , Kutschan U , Meiswinkel N , Schulte-Hubbert B , Ewig S , Kolditz M . 2018. Admission lactate predicts poor prognosis independently of the CRB/CURB-65 scores in community-acquired pneumonia. Clin Microbiol Infect 24:306. doi:10.1016/j.cmi.2017.07.007 28710027

[19] Yang Z , Zhang S , Tang Y-P , Zhang S , Xu D-Q , Yue S-J , Liu Q-L . 2022. Clinical characteristics, transmissibility, pathogenicity, susceptible populations, and re-infectivity of prominent COVID-19 variants. Aging Dis 13:402–422. doi:10.14336/AD.2021.1210 35371608 PMC8947836

[20] Lindsley AW , Schwartz JT , Rothenberg ME . 2020. Eosinophil responses during COVID-19 infections and Coronavirus vaccination. J Allergy Clin Immunol 146:1–7. doi:10.1016/j.jaci.2020.04.021 32344056 PMC7194727

[21] Yan B , Yang J , Xie Y , Tang X . 2021. Relationship between blood eosinophil levels and COVID-19 mortality. World Allergy Organ J 14:100521. doi:10.1016/j.waojou.2021.100521 33589865 PMC7877210

[22] Zhou X , Chen D , Wang L , Zhao Y , Wei L , Chen Z , Yang B . 2020. Low serum calcium: a new, important indicator of COVID-19 patients from mild/moderate to severe/critical. Biosci Rep 40:BSR20202690. doi:10.1042/BSR20202690 33252122 PMC7755121

[23] Bajwa H , Riaz Y , Ammar M , Farooq S , Yousaf A . 2020. The dilemma of renal involvement in COVID-19: a systematic review. Cureus 12:e8632. doi:10.7759/cureus.8632 32685301 PMC7364426

[24] Ferastraoaru D , Hudes G , Jerschow E , Jariwala S , Karagic M , de Vos G , Rosenstreich D , Ramesh M . 2021. Eosinophilia in asthma patients is protective against severe COVID-19 illness. J Allergy Clin Immunol Pract 9:1152–1162. doi:10.1016/j.jaip.2020.12.045 33495097 PMC7826039

[25] Luth EA , Pan CX , Viola M , Prigerson HG . 2021. Dementia and early do-not-resuscitate orders associated with less intensive of end-of-life care: a retrospective cohort study. Am J Hosp Palliat Care 38:1417–1425. doi:10.1177/1049909121989020 33467864 PMC8289944

[26] Barnato AE , Birkmeyer JD , Skinner JS , O’Malley AJ , Birkmeyer NJO . 2022. Treatment intensity and mortality among COVID-19 patients with dementia: a retrospective observational study. J Am Geriatr Soc 70:40–48. doi:10.1111/jgs.17463 34480354 PMC8742761

[27] Vaid A , Somani S , Russak AJ , De Freitas JK , Chaudhry FF , Paranjpe I , Johnson KW , Lee SJ , Miotto R , Richter F , Zhao S , Beckmann ND , Naik N , Kia A , Timsina P , Lala A , Paranjpe M , Golden E , Danieletto M , Singh M , Meyer D , O’Reilly PF , Huckins L , Kovatch P , Finkelstein J , Freeman RM , Argulian E , Kasarskis A , Percha B , Aberg JA , Bagiella E , Horowitz CR , Murphy B , Nestler EJ , Schadt EE , Cho JH , Cordon-Cardo C , Fuster V , Charney DS , Reich DL , Bottinger EP , Levin MA , Narula J , Fayad ZA , Just AC , Charney AW , Nadkarni GN , Glicksberg BS . 2020. Machine learning to predict mortality and critical events in a cohort of patients with COVID-19 in New York city: model development and validation. J Med Internet Res 22:e24018. doi:10.2196/24018 33027032 PMC7652593

[28] Vaid A , Chan L , Chaudhary K , Jaladanki SK , Paranjpe I , Russak A , Kia A , Timsina P , Levin MA , He JC , Böttinger EP , Charney AW , Fayad ZA , Coca SG , Glicksberg BS , Nadkarni GN , MSCIC . 2021. Predictive approaches for acute dialysis requirement and death in COVID-19. Clin J Am Soc Nephrol 16:1158–1168. doi:10.2215/CJN.17311120 34031183 PMC8455052

[29] Vaid A , Jaladanki SK , Xu J , Teng S , Kumar A , Lee S , Somani S , Paranjpe I , De Freitas JK , Wanyan T , Johnson KW , Bicak M , Klang E , Kwon YJ , Costa A , Zhao S , Miotto R , Charney AW , Böttinger E , Fayad ZA , Nadkarni GN , Wang F , Glicksberg BS . 2021. Federated learning of electronic health records to improve mortality prediction in hospitalized patients with COVID-19: machine learning approach. JMIR Med Inform 9:e24207. doi:10.2196/24207 33400679 PMC7842859

[30] Barda N , Riesel D , Akriv A , Levy J , Finkel U , Yona G , Greenfeld D , Sheiba S , Somer J , Bachmat E , Rothblum GN , Shalit U , Netzer D , Balicer R , Dagan N . 2020. Developing a COVID-19 mortality risk prediction model when individual-level data are not available. Nat Commun 11:4439. doi:10.1038/s41467-020-18297-9 32895375 PMC7477233

[31] Fernandes FT , de Oliveira TA , Teixeira CE , Batista A de M , Dalla Costa G , Chiavegatto Filho ADP . 2021. A multipurpose machine learning approach to predict COVID-19 negative prognosis in São Paulo, Brazil. Sci Rep 11:3343. doi:10.1038/s41598-021-82885-y 33558602 PMC7870665

[32] Feng Z , Yu Q , Yao S , Luo L , Zhou W , Mao X , Li J , Duan J , Yan Z , Yang M , Tan H , Ma M , Li T , Yi D , Mi Z , Zhao H , Jiang Y , He Z , Li H , Nie W , Liu Y , Zhao J , Luo M , Liu X , Rong P , Wang W . 2020. Early prediction of disease progression in COVID-19 pneumonia patients with chest CT and clinical characteristics. Nat Commun 11:4968. doi:10.1038/s41467-020-18786-x 33009413 PMC7532528

[33] Liang W , Liang H , Ou L , Chen B , Chen A , Li C , Li Y , Guan W , Sang L , Lu J , Xu Y , Chen G , Guo H , Guo J , Chen Z , Zhao Y , Li S , Zhang N , Zhong N , He J , China Medical Treatment Expert Group for COVID-19 . 2020. Development and validation of a clinical risk score to predict the occurrence of critical illness in hospitalized patients with COVID-19. JAMA Intern Med 180:1081–1089. doi:10.1001/jamainternmed.2020.2033 32396163 PMC7218676

[34] Mahdavi M , Choubdar H , Zabeh E , Rieder M , Safavi-Naeini S , Jobbagy Z , Ghorbani A , Abedini A , Kiani A , Khanlarzadeh V , Lashgari R , Kamrani E . 2021. A machine learning based exploration of COVID-19 mortality risk. PLoS One 16:e0252384. doi:10.1371/journal.pone.0252384 34214101 PMC8253432

[35] Ning W , Lei S , Yang J , Cao Y , Jiang P , Yang Q , Zhang J , Wang X , Chen F , Geng Z , Xiong L , Zhou H , Guo Y , Zeng Y , Shi H , Wang L , Xue Y , Wang Z . 2020. Open resource of clinical data from patients with pneumonia for the prediction of COVID-19 outcomes via deep learning. Nat Biomed Eng 4:1197–1207. doi:10.1038/s41551-020-00633-5 33208927 PMC7723858

[36] Booth AL , Abels E , McCaffrey P . 2021. Development of a prognostic model for mortality in COVID-19 infection using machine learning. Mod Pathol 34:522–531. doi:10.1038/s41379-020-00700-x 33067522 PMC7567420

[37] Naseem M , Arshad H , Hashmi SA , Irfan F , Ahmed FS . 2021. Predicting mortality in SARS-COV-2 (COVID-19) positive patients in the inpatient setting using a novel deep neural network. Int J Med Inform 154:104556. doi:10.1016/j.ijmedinf.2021.104556 34455118 PMC8378987

[38] Estiri H , Strasser ZH , Klann JG , Naseri P , Wagholikar KB , Murphy SN . 2021. Predicting COVID-19 mortality with electronic medical records. NPJ Digit Med 4:15. doi:10.1038/s41746-021-00383-x 33542473 PMC7862405

[39] Bennett TD , Moffitt RA , Hajagos JG , Amor B , Anand A , Bissell MM , Bradwell KR , Bremer C , Byrd JB , Denham A , DeWitt PE , Gabriel D , Garibaldi BT , Girvin AT , Guinney J , Hill EL , Hong SS , Jimenez H , Kavuluru R , Kostka K , Lehmann HP , Levitt E , Mallipattu SK , Manna A , McMurry JA , Morris M , Muschelli J , Neumann AJ , Palchuk MB , Pfaff ER , Qian Z , Qureshi N , Russell S , Spratt H , Walden A , Williams AE , Wooldridge JT , Yoo YJ , Zhang XT , Zhu RL , Austin CP , Saltz JH , Gersing KR , Haendel MA , Chute CG , National COVID Cohort Collaborative (N3C) Consortium . 2021. Clinical characterization and prediction of clinical severity of SARS-Cov-2 infection among US adults using data from the US national COVID cohort collaborative. JAMA Netw Open 4:e2116901. doi:10.1001/jamanetworkopen.2021.16901 34255046 PMC8278272

[40] Dar M , Swamy L , Gavin D , Theodore A . 2021. Mechanical-ventilation supply and options for the COVID-19 pandemic leveraging all available resources for a limited resource in a crisis. Ann Am Thorac Soc 18:408–416. doi:10.1513/AnnalsATS.202004-317CME 33202144 PMC7919160

[41] Kamath PS , Wiesner RH , Malinchoc M , Kremers W , Therneau TM , Kosberg CL , D’Amico G , Dickson ER , Kim WR . 2001. A model to predict survival in patients with end-stage liver disease. Hepatology 33:464–470. doi:10.1053/jhep.2001.22172 11172350

[42] Gage BF , Waterman AD , Shannon W , Boechler M , Rich MW , Radford MJ . 2001. Validation of clinical classification schemes for predicting stroke. JAMA 285:2864. doi:10.1001/jama.285.22.2864 11401607

[43] Ahmad FB , Cisewski JA , Xu J , Anderson RN . 2023. Provisional mortality data—United States, 2022. MMWR Morb Mortal Wkly Rep 72:488–492. doi:10.15585/mmwr.mm7218a3 37141156 PMC10168603

[44] Alexander MP , Mangalaparthi KK , Madugundu AK , Moyer AM , Adam BA , Mengel M , Singh S , Herrmann SM , Rule AD , Cheek EH , Herrera Hernandez LP , Graham RP , Aleksandar D , Aubry M-C , Roden AC , Hagen CE , Quinton RA , Bois MC , Lin PT , Maleszewski JJ , Cornell LD , Sethi S , Pavelko KD , Charlesworth J , Narasimhan R , Larsen CP , Rizza SA , Nasr SH , Grande JP , McKee TD , Badley AD , Pandey A , Taner T . 2021. Acute kidney injury in severe COVID-19 has similarities to sepsis-associated kidney injury. Mayo Clin Proc 96:2561–2575. doi:10.1016/j.mayocp.2021.07.001 34425963 PMC8279954

[45] Ferlicot S , Just P-A , Compérat E , Rouleau E , Tissier F , Vaessen C , Richard S . 2021. Clear cell and papillary renal cell carcinomas in hereditary papillary renal cell carcinoma (HPRCC) syndrome: a case report. Diagn Pathol 16:107. doi:10.1186/s13000-021-01170-8 34801057 PMC8606058

[46] Vassiliou AG , Tsipilis S , Keskinidou C , Vrettou CS , Jahaj E , Gallos P , Routsi C , Orfanos SE , Kotanidou A , Dimopoulou I . 2022. Lactate and lactate-to-pyruvate ratio in critically ill COVID-19 patients: a pilot study. J Pers Med 12:171. doi:10.3390/jpm12020171 35207659 PMC8880262

[47] Wool GD , Miller JL . 2021. The impact of COVID-19 disease on platelets and coagulation. Pathobiology 88:15–27. doi:10.1159/000512007 33049751 PMC7649697

[48] Terpos E , Ntanasis-Stathopoulos I , Elalamy I , Kastritis E , Sergentanis TN , Politou M , Psaltopoulou T , Gerotziafas G , Dimopoulos MA . 2020. Hematological findings and complications of COVID-19. Am J Hematol 95:834–847. doi:10.1002/ajh.25829 32282949 PMC7262337

[49] Henry BM , Benoit JL , Benoit S , Pulvino C , Berger BA , Olivera M de , Crutchfield CA , Lippi G . 2020. Red blood cell distribution width (RDW) predicts COVID-19 severity: a prospective, observational study from the cincinnati SARS-Cov-2 emergency department cohort. Diagnostics 10:618. doi:10.3390/diagnostics10090618 32825629 PMC7554711

[50] Wang C , Zhang H , Cao X , Deng R , Ye Y , Fu Z , Gou L , Shao F , Li J , Fu W , Zhang X , Ding X , Xiao J , Wu C , Li T , Qi H , Li C , Lu Z . 2020. Red cell distribution width (RDW): a Prognostic indicator of severe COVID-19. Ann Transl Med 8:1230. doi:10.21037/atm-20-6090 33178762 PMC7607068

[51] Soni M , Gopalakrishnan R . 2021. Significance of RDW in predicting mortality in COVID-19—an analysis of 622 cases. Int J Lab Hematol 43:O221–O223. doi:10.1111/ijlh.13526 33774907 PMC8250958

[52] Abidi K , Khoudri I , Belayachi J , Madani N , Zekraoui A , Zeggwagh AA , Abouqal R . 2008. Eosinopenia is a reliable marker of sepsis on admission to medical intensive care units. Crit Care 12:R59. doi:10.1186/cc6883 18435836 PMC2447615

[53] Xia Z . 2020. Eosinopenia as an early diagnostic marker of COVID-19 at the time of the epidemic. EClinicalMedicine 23:100398. doi:10.1016/j.eclinm.2020.100398 32572392 PMC7299848

[54] Lombardi C , Bagnasco D , Passalacqua G . 2022. COVID-19, eosinophils, and biologicals for severe asthma. Front Allergy 3:859376. doi:10.3389/falgy.2022.859376 35769563 PMC9234863

[55] Ito A , Ishida T , Nakanishi Y , Kobe H , Tokioka F . 2023. Eosinopenia is associated with adverse outcomes after COVID-19 infection: a perspective from Japan. Respirology 28:677–680. doi:10.1111/resp.14509 37105899

[56] Gatti M , Calandri M , Biondo A , Geninatti C , Piatti C , Ruggirello I , Santonocito A , Varello S , Bergamasco L , Bironzo P , Boccuzzi A , Brazzi L , Caironi P , Cardinale L , Cavallo R , Riccardini F , Limerutti G , Veltri A , Fonio P , Faletti R . 2022. Emergency room comprehensive assessment of demographic, radiological, laboratory and clinical data of patients with COVID-19: determination of its prognostic value for in-hospital mortality. Intern Emerg Med 17:205–214. doi:10.1007/s11739-021-02669-0 33683539 PMC7938271

[57] Zhang H , Shi T , Wu X , Zhang X , Wang K , Bean D , Dobson R , Teo JT , Sun J , Zhao P , Li C , Dhaliwal K , Wu H , Li Q , Guthrie B . 2020. Risk prediction for poor outcome and death in hospital in-patients with COVID-19: derivation in Wuhan, China and external validation in London, UK. Public and global health. doi:10.1101/2020.04.28.20082222

[58] Costa VDO , Nicolini EM , da Costa BMA , Ferreira VHP , Tonisi AJR , Machado NM , Moura M de A , Montessi J , de Castro Ferreira L , Campos RL , Costa PM , Campos MA . 2021. Sociodemographic, laboratory, image data and predictors of gravity risk in patients with COVID-19. PLoS One 16:e0256331. doi:10.1371/journal.pone.0256331 34411145 PMC8375972

[59] Kim JH . 2019. Multicollinearity and misleading statistical results. Korean J Anesthesiol 72:558–569. doi:10.4097/kja.19087 31304696 PMC6900425

[60] Banoei MM , Dinparastisaleh R , Zadeh AV , Mirsaeidi M . 2021. Machine-learning-based COVID-19 mortality prediction model and identification of patients at low and high risk of dying. Crit Care 25:328. doi:10.1186/s13054-021-03749-5 34496940 PMC8424411

[61] Kar S , Chawla R , Haranath SP , Ramasubban S , Ramakrishnan N , Vaishya R , Sibal A , Reddy S . 2021. Multivariable mortality risk prediction using machine learning for COVID-19 patients at admission (AICOVID). Sci Rep 11:12801. doi:10.1038/s41598-021-92146-7 34140592 PMC8211710

[62] Lundberg S , Lee S-I . 2017. A unified approach to interpreting model predictions. arXiv. doi:https://arxiv.org/abs/1705.07874

[63] Haendel MA , Chute CG , Bennett TD , Eichmann DA , Guinney J , Kibbe WA , Payne PRO , Pfaff ER , Robinson PN , Saltz JH , Spratt H , Suver C , Wilbanks J , Wilcox AB , Williams AE , Wu C , Blacketer C , Bradford RL , Cimino JJ , Clark M , Colmenares EW , Francis PA , Gabriel D , Graves A , Hemadri R , Hong SS , Hripscak G , Jiao D , Klann JG , Kostka K , Lee AM , Lehmann HP , Lingrey L , Miller RT , Morris M , Murphy SN , Natarajan K , Palchuk MB , Sheikh U , Solbrig H , Visweswaran S , Walden A , Walters KM , Weber GM , Zhang XT , Zhu RL , Amor B , Girvin AT , Manna A , Qureshi N , Kurilla MG , Michael SG , Portilla LM , Rutter JL , Austin CP , Gersing KR , N3C Consortium . 2021. The National COVID cohort collaborative (N3C): rationale, design, infrastructure, and deployment. J Am Med Inform Assoc 28:427–443. doi:10.1093/jamia/ocaa196 32805036 PMC7454687

[64] National COVID Cohort Collaborative . 2023. GitHub. https://github.com/National-COVID-Cohort-Collaborative

[65] Pedregosa F , Gramfort A , Michel V , Thirion B , Grisel O , Blondel M , Nothman J , Louppe G , Prettenhofer P , Weiss R , Dubourg V , Vanderplas J , Passos A , Cournapeau D , Brucher M , Perrot M , Duchesnay E . 2018. Scikit-learn, . In Machine learning in python

[66] Chen T , Guestrin C . 2022. Xgboost. ACM, New York, USA.

[67] Harris CR , Millman KJ , van der Walt SJ , Gommers R , Virtanen P , Cournapeau D , Wieser E , Taylor J , Berg S , Smith NJ , Kern R , Picus M , Hoyer S , van Kerkwijk MH , Brett M , Haldane A , Del Río JF , Wiebe M , Peterson P , Gérard-Marchant P , Sheppard K , Reddy T , Weckesser W , Abbasi H , Gohlke C , Oliphant TE . 2020. Array programming with numpy. Nature 585:357–362. doi:10.1038/s41586-020-2649-2 32939066 PMC7759461

[68] McKinney W . 2010. Data structures for statistical computing in python Python in Science Conference; Austin, Texas: . doi:10.25080/Majora-92bf1922-00a

[69] Virtanen P , Gommers R , Oliphant TE , Haberland M , Reddy T , Cournapeau D , Burovski E , Peterson P , Weckesser W , Bright J , van der Walt SJ , Brett M , Wilson J , Millman KJ , Mayorov N , Nelson ARJ , Jones E , Kern R , Larson E , Carey CJ , Polat İ , Feng Y , Moore EW , VanderPlas J , Laxalde D , Perktold J , Cimrman R , Henriksen I , Quintero EA , Harris CR , Archibald AM , Ribeiro AH , Pedregosa F , van Mulbregt P , Vijaykumar A , Bardelli AP , Rothberg A , Hilboll A , Kloeckner A , Scopatz A , Lee A , Rokem A , Woods CN , Fulton C , Masson C , Häggström C , Fitzgerald C , Nicholson DA , Hagen DR , Pasechnik DV , Olivetti E , Martin E , Wieser E , Silva F , Lenders F , Wilhelm F , Young G , Price GA , Ingold G-L , Allen GE , Lee GR , Audren H , Probst I , Dietrich JP , Silterra J , Webber JT , Slavič J , Nothman J , Buchner J , Kulick J , Schönberger JL , de Miranda Cardoso JV , Reimer J , Harrington J , Rodríguez JLC , Nunez-Iglesias J , Kuczynski J , Tritz K , Thoma M , Newville M , Kümmerer M , Bolingbroke M , Tartre M , Pak M , Smith NJ , Nowaczyk N , Shebanov N , Pavlyk O , Brodtkorb PA , Lee P , McGibbon RT , Feldbauer R , Lewis S , Tygier S , Sievert S , Vigna S , Peterson S , More S , Pudlik T , Oshima T , Pingel TJ , Robitaille TP , Spura T , Jones TR , Cera T , Leslie T , Zito T , Krauss T , Upadhyay U , Halchenko YO , Vázquez-Baeza Y , SciPy 1.0 Contributors . 2020. Scipy 1.0: fundamental algorithms for scientific computing in python. Nat Methods 17:261–272. doi:10.1038/s41592-020-0772-5 32015543 PMC7056644

[70] Hunter JD . 2007. Matplotlib: a 2d graphics environment. Comput Sci Eng 9:90–95. doi:10.1109/MCSE.2007.55

[71] Waskom M . 2021. Seaborn: statistical data visualization. JOSS 6:3021. doi:10.21105/joss.03021

[72] Pollard TJ , Johnson AEW , Raffa JD , Mark RG . 2018. Tableone: an open source python package for producing summary statistics for research papers. J Am Med Inform Assoc 1:26–31. doi:10.1093/jamiaopen/ooy012 PMC6951995 31984317

