# Supplementary material for: Predicting COVID-19 prognosis in hospitalized patients based on early status
Source: mBio. 2023 Sep 8;14(5):e01508-23. doi: 10.1128/mbio.01508-23 (PMC10653946; doi:10.1128/mbio.01508-23)
Supplement: Fig. S1 — Study design. [file mbio.01508-23-s0001.docx]

**Supplemental Figure 1*.* Study Design**

**
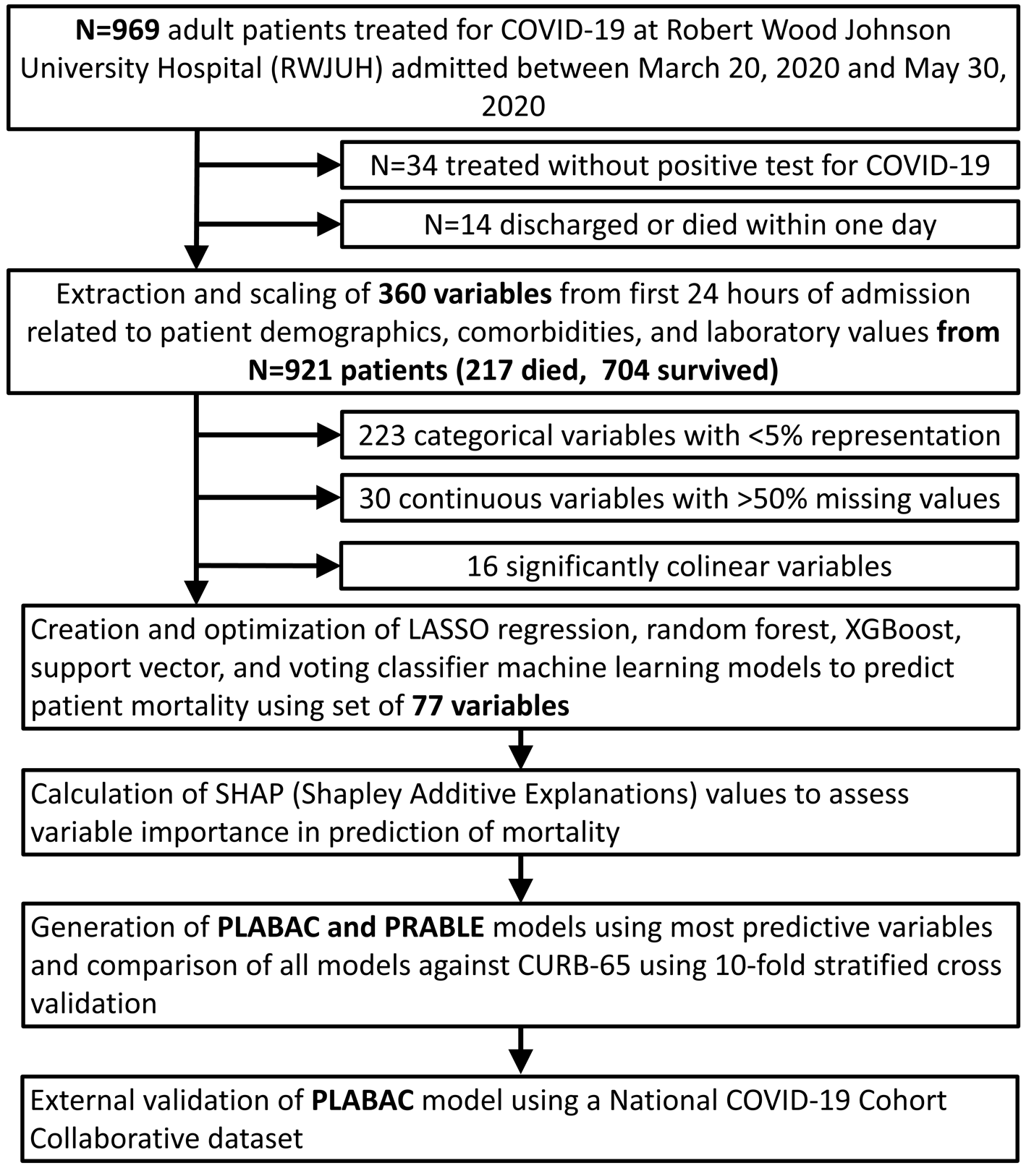
**

Abbreviations: RWJUH, Robert Wood Johnson University Hospital; LASSO, least absolute shrinkage and selection operator; SHAP, Shapley Additive exPlanations
