# Supplementary material for: Predicting COVID-19 prognosis in hospitalized patients based on early status
Source: mBio. 2023 Sep 8;14(5):e01508-23. doi: 10.1128/mbio.01508-23 (PMC10653946; doi:10.1128/mbio.01508-23)
Supplement: Fig. S2 — Feature importance identified by SHAP values for the prediction of intubation by the voting classifier. [file mbio.01508-23-s0002.docx]

**Supplemental Figure 2. Feature importance identified by SHAP values for the prediction of intubation by the voting classifier**


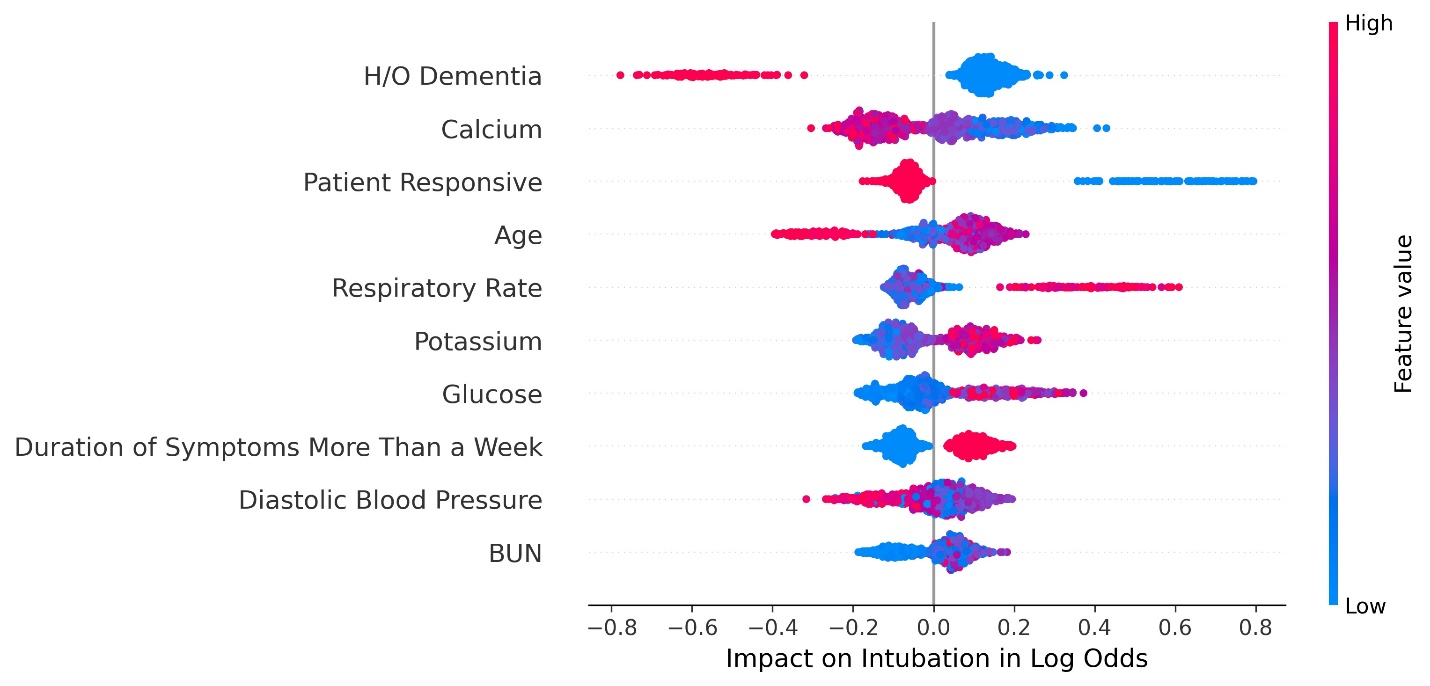


Each point on the plot is a patient’s value for the specified variable, in ranked feature importance for the voting classifier for mortality prediction. The numerical feature values are shown on a red (high)-blue (low) scale. For binary variables, (e.g. patient responsive and h/o dementia), red indicates trait presence and blue indicates absence. Features are shown in descending order of average absolute importance. Impact on model output is shown as log odds for mortality.

Abbreviations: H/O, history of; BUN, blood urea nitrogen.
