# Supplementary material for: Predicting COVID-19 prognosis in hospitalized patients based on early status
Source: mBio. 2023 Sep 8;14(5):e01508-23. doi: 10.1128/mbio.01508-23 (PMC10653946; doi:10.1128/mbio.01508-23)
Supplement: Fig. S3 — Feature importance identified by SHAP values for the prediction of ICU admission by XGBoost. [file mbio.01508-23-s0003.docx]

**Supplemental Figure 3. Feature importance identified by SHAP values for the prediction of ICU admission by XGBoost**


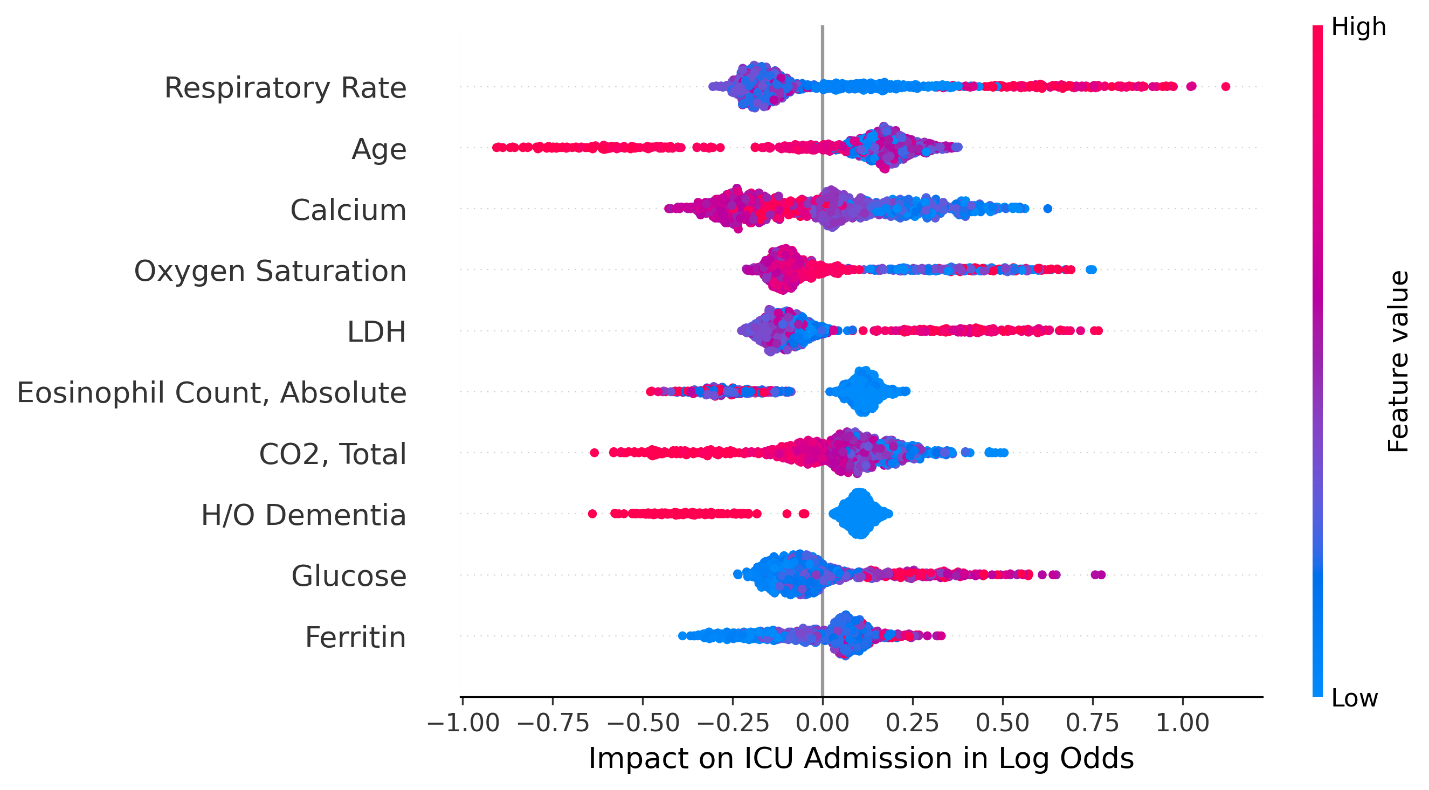


Each point on the plot is a patient’s value for the specified variable, in ranked feature importance for the voting classifier for mortality prediction. The numerical feature values are shown on a red (high)-blue (low) scale. For binary variables, (e.g. dementia), red indicates trait presence and blue indicates absence. Features are shown in descending order of average absolute importance. Impact on model output is shown as log odds for mortality.

Abbreviations: LDH, lactate dehydrogenase; H/O, history of.
