# Supplementary material for: Predicting COVID-19 prognosis in hospitalized patients based on early status
Source: mBio. 2023 Sep 8;14(5):e01508-23. doi: 10.1128/mbio.01508-23 (PMC10653946; doi:10.1128/mbio.01508-23)
Supplement: Fig. S4 — Importance of dementia in the prediction of mortality, intubation, and ICU admission. [file mbio.01508-23-s0004.docx]

**Supplemental Figure 4. Importance of dementia in the prediction of mortality, intubation, and ICU admission**

**4a. Mortality**


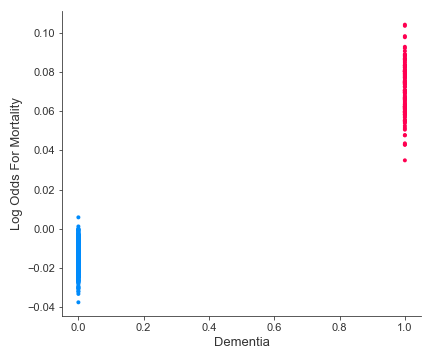


**4b. Intubation**


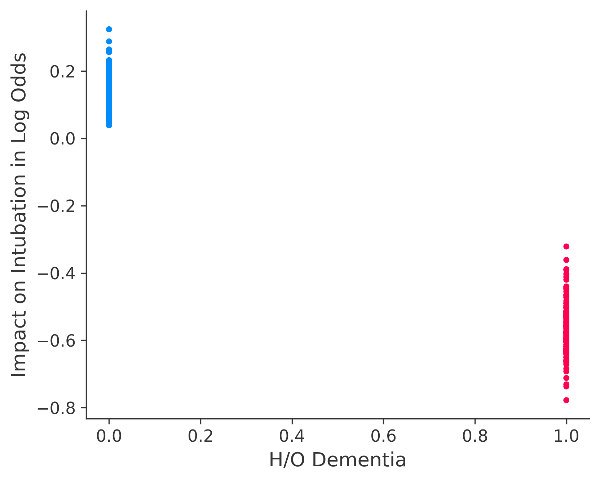


**4c. ICU Admission**


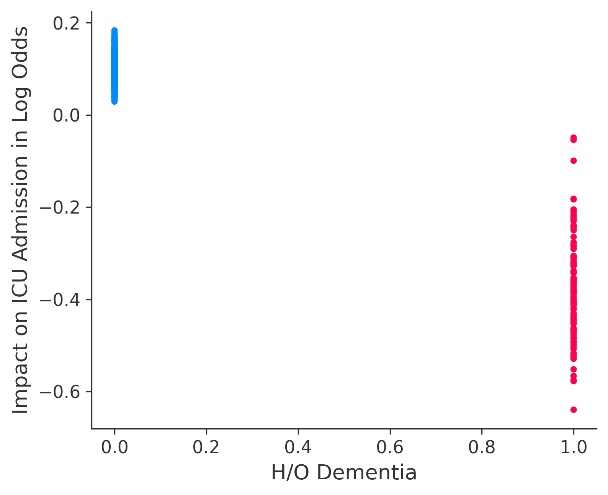


SHAP values for dementia for our top models for mortality, intubation, and ICU admission. Y axis is the impact of a value with respect to model output expressed in log odds, in which a more positive number is associated with higher risk, and the X axis is the actual value of the variable where 0 is no history dementia and 1 is history of dementia.

Abbreviations: H/O, history of.
