# Supplementary material for: Predicting COVID-19 prognosis in hospitalized patients based on early status
Source: mBio. 2023 Sep 8;14(5):e01508-23. doi: 10.1128/mbio.01508-23 (PMC10653946; doi:10.1128/mbio.01508-23)
Supplement: Table S1 — Binning methods for symptoms. [file mbio.01508-23-s0005.docx]

**Supplemental Table 1. Binning Methods for Symptoms**

| **Category** | **Symptoms included** |
| --- | --- |
| Cardiovascular | Cardiac Arrest, Chest Pain, Chest Tightness, Palpitations, Unilateral Leg Swelling |
| Constitutional | Chills, Diaphoresis, Dizziness/Lightheadedness, Failure to Thrive, Fatigue, Fever, Lethargy, Lymphadenopathy, Malaise, Myalgia/Arthralgia, Weakness |
| Gastrointestinal | Abdominal Distension, Abdominal Pain, Anorexia/Poor Appetite, Bloody/Dark Black Stools, Bowel Incontinence, Constipation, Diarrhea, Nausea, Odynophagia, Reflux, Vomiting |
| Neuro-Psychiatric | Ageusia, Altered Mental Status, Anosmia, Aphasia, Conjunctival Injection, Conjunctivitis, Dysarthria, Facial Weakness, Headache, Numbness/Loss of Sensation, Paresthesia, Photophobia, Psychiatric Disturbance, Scleral Injection, Seizure, Syncope |
| Respiratory | Chest Congestion, Cough (dry, productive), Dyspnea/Shortness of Breath, Hemoptysis, Nasal Congestion, Orthopnea, Pain with Cough, Pleuritic Chest Pain, Sore Throat, Tonsillitis, Wheezing |
