# Supplementary material for: Predicting COVID-19 prognosis in hospitalized patients based on early status
Source: mBio. 2023 Sep 8;14(5):e01508-23. doi: 10.1128/mbio.01508-23 (PMC10653946; doi:10.1128/mbio.01508-23)
Supplement: Table S2 — Binning methods for comorbidities. [file mbio.01508-23-s0006.docx]

**Supplemental Table 2. Binning Methods for Comorbidities**

| **Category** | **Diseases Included** |
| --- | --- |
| Arrythmia | Atrial Fibrillation, Atrial Flutter, Atrioventricular Nodal Reentry Tachycardia, AV Block, Bradycardia, Brugada Syndrome, Cardiac Arrest, Heart Block Pacemaker or Defibrillator, Prolonged Qtc, Sick Sinus Syndrome, Ventricular Fibrillation, Ventricular Tachycardia |
| Coronary Artery Disease or MI History | Acute Coronary Syndrome, Arteriosclerotic Heart Disease, Coronary Artery Bypass Graft, Coronary Artery Disease, Coronary Stent Placement, Myocardial Infarction, Percutaneous Coronary Intervention |
| DVT/PE | Arterial Occlusion Of Brachial Artery, Deep Vein Thrombosis, Inferior Vena Cava Filter, Pulmonary Embolism |
| Heart Failure | Cardiomyopathy, Chronic Heart Failure, Diastolic Dysfunction, Dilated Cardiomyopathy, Heart Failure, Idiopathic Cardiomyopathy, Ischemic Cardiomyopathy, Left Ventricular Systolic Dysfunction, Non-Ischemic Cardiomyopathy, Systolic Cardiomyopathy, Viral Cardiomyopathy |
| Hypertension | Hypertension, Preeclampsia |
| Cerebrovascular Disease | Anoxic Brain Injury, Basal Ganglia Infarct, Brain Aneurysm, Cerebellar Artery Stent-Graft, Cerebellar Hemorrhage, Cerebrovascular Accident / Stroke, Chronic Infarct on Head CT, Intracerebral Aneurysms, Intracranial Hypertension, Intraparenchymal Hemorrhage, Subarachnoid Hemorrhage, Subdural Hematoma, Transient Ischemic Attack, Wallenberg’s Syndrome |
| Genitourinary Disease | Atypical Hyperplasia Of Endometrium, Benign Prostate Hyperplasia, Bladder Implant, Cervical Implant, Chronic Pelvis Abscess, Dysfunctional Uterine Bleeding, Epididymitis / Orchitis, Fournier Gangrene, Gangrene Of the Penis, HSV Genital, Hysterectomy, Menorrhagia, Ovarian Cyst, Overactive Bladder, Penile Syringoma, Perianal Fistula, Polycystic Ovarian Syndrome, Prostatitis, Recurrent UTIs, Transurethral Resection of the Prostate, Ureteral Colic, Ureteral Strictures, Urinary Bladder Stones, Urinary Incontinence, Urinary Retention, Uterine Fibroid |
| GI Disease | Achalasia, Alcohol Induced Chronic Pancreatitis, Barrett’s Esophagus, Candida Esophagitis/Gastritis, Cholecystectomy, Cholecystitis, Choledocholithiasis, Cholelithiasis, Chronic Constipation, Chronic Dysphagia, Colitis, Cyclic Vomiting Syndrome, Dieulafoy's Lesion Gastritis, Diverticulitis, Diverticulosis, Duodenum Ulcer, Endoscopic Retrograde Cholangiopancreatography With Sphincterotomy And Stent, Esophageal Ulcers, Esophagitis, Gallbladder Disease, Gallbladder Sludge, Gallstone Pancreatitis, Gastric Diverticulum, Gastric Ulcer, Gastritis, Gastroesophageal Reflux Disease, Gastrointestinal Bleed, Gastrointestinal Disease, Hartmann's Operation, Hemorrhoids, Hernia, Hernia Repair, Ileal Conduit, Ileostomy, Intraabdominal Abscesses, Intussusception, Irritable Bowel Syndrome, Multiple Bowel Obstructions, Pancreatic Pseudocyst, Pancreatitis, Peptic Ulcer, Peptic Ulcer Disease, Perforated Cholecystoduodenal Fistula, Perigastric Abscess, Pouchitis, Rectal Prolapse, Recurrent C. Diff. Colitis, Reflux, Reflux Esophagitis, Sigmoid Colostomy Stricture, Small Bowel Obstruction, Stercoral Ulcers, Surgery For Colon Infection, Ulcerative Colitis, Ulcerative Proctitis |
| Anemia | Alpha-Thalassemia Anemia, Anemia, Anemia of Chronic Disease, Anemia of Renal Disease, Autoimmune Hemolytic Anemia, Beta-Thalassemia, Beta-Thalassemia Sickle Cell Trait, Chronic Anemia, Hbsc Hemoglobinopathy, Iron Deficiency Anemia, Microcytic Anemia, Normocytic Anemia, Pernicious Anemia, Sickle Cell Disease, Thalassemia |
| Malignancy | Acute Lymphocytic Leukemia, Acute Myelocytic Leukemia, Adenocarcinoma, Atypical Meningioma, Basal Cell Carcinoma, B-Cell Acute Lymphocytic Leukemia, B-Cell Lymphoma, Biliary Ductal Carcinoma, Bladder Cancer, Brain Mass, Brain Tumor, Breast Cancer, Bronchogenic Carcinoma, Burkitt's Lymphoma, Calvarial Tumor Resection, Cervical Cancer, Chronic Lymphocytic Leukemia, Chronic Myeloid Leukemia, Colon Cancer, Complex Atypical Hyperplasia Of Endometrium S/P Hysterectomy, Diencephalon Tumor, Diffuse Large B-Cell Lymphoma, Endometrial Cancer, Familial Adenomatous Polyposis, Gastric Cancer, Gynecological Cancer, Hodgkin’s Lymphoma, Intraductal Papillary Mucinous Neoplasm, Intrathoracic Tumor, Laryngeal Squamous Cell Carcinoma, Liver Cancer, Lung Cancer, Lung Mass S/P Chemotherapy And Radiotherapy, Lymphoma, Malignant Neoplasm Of The Tongue And Pharynx, Malignant Pleural Effusion, Melanoma, Metastatic Spinal Compression Fractures, Monoclonal Gammopathy Of Undetermined Significance, Multiple Myeloma, Myelodysplastic Syndromes, Nasopharyngeal Cancer, Non-Hodgkin’s Lymphoma, Non-Small Cell Lung Cancer, Ovarian Cancer, Pancreatic Cancer, Pancreatic/Biliary Adenocarcinoma, Pancytopenia S/P Recent Chemotherapy, Papillary Thyroid Cancer, Penile Cancer In Situ, Pharyngeal Carcinoma, Prostate Cancer, Rectal Cancer, Renal Cancer, Renal Carcinoma, Renal Cell Carcinoma, Renal Mass With Resection, Sigmoid Colon Cancer Adenocarcinoma, Skin Cancer, Squamous Cell Carcinoma, Thyroid Cancer, Urothelial Cancer, Uterine Cancer, Vulvar Cancer |
| Diabetes | Diabetes, Diabetes Mellitus, Diabetes Mellitus Type 1 / Insulin Dependent Diabetes Mellitus, Diabetes Mellitus Type 2 / Non-Insulin Dependent Diabetes Mellitus, Diabetic Foot Ulcers, Diabetic Neuropathy, Diabetic Retinopathy, Gestational Diabetes Mellitus, New Onset Diabetes / Newly Diagnosed Diabetes Mellitus |
| Hyperlipidemia | Dyslipidemia, Hypercholesterolemia, Hyperlipidemia, Hypertriglyceridemia |
| Hypothyroidism | Hashimoto's Thyroiditis, Hypothyroidism |
| Musculoskeletal Disease | Arthritis, Arthroplasty, Avascular Necrosis Of Hip, Back Surgery, Bilateral Inguinal Hernia S/P Repair, Bunionectomy, Calcium Pyrophosphate Deposition Disease, Chronic Back Pain, Compression Fracture, Gout, Hemiarthroplasty, Herniation Of Nucleus Pulposus, Hip Fracture, Hip Replacement, Knee Placement, Kyphosis, Laminectomy, Lower Extremity Amputation, Lumbar Radiculopathy, Multiple Orthopedic Surgeries, Olecranon Bursitis / Cellulitis, Open Reduction And Internal Fixation, Osteoarthritis / Degenerative Joint Disease, Osteopenia, Osteoporosis, Rhabdomyolysis, Rib Fractures, Sacral Decubitus Ulcer, Sciatica, Scoliosis, Spina Bifida, Spinal Destruction, Spinal Stenosis, Spine Surgery, Spondylosis |
| Dementia | Alzheimer’s Disease, Dementia, Lewy Body Dementia, Parkinson's Disease |
| Other Neurological Disease | Aphasia, Arnold-Chiari Malformation, Ataxia, Bell's Palsy, Central Canal Stenosis / Foraminal Narrowing, Cerebellar Cavernoma, Chronic Fatigue Syndrome, Encephalitis, Encephalomalacia, Encephalopathy, Essential Tremor, Fibromyalgia, Guillain Barre Syndrome, Hallervorden-Spatz Disease, Head Injury / Traumatic Brain Injury, Hydrocephalus, Idiopathic Myeloneuropathy, Korsakoff’s Disease, Meningioma S/P Resection, Migraines, Multiple Sclerosis, Muscular Weakness Secondary To Polio, Neurogenic Bladder, Neurosyphilis, Paraplegia, Polio, Restless Leg Syndrome, Sacral Neuromodulatory Implant, Spinal Cord Compression, Spinal Cord Injury, Vocal Cord Paralysis |
| Anxiety | Generalized Anxiety Disorder |
| Depression | Major Depressive Disorder, Past Suicidal Ideation, and Suicide Attempt |
| COPD | Emphysema, Bronchitis, and Unspecified COPD |
| Other Pulmonary Disease | Bronchiectasis, Chronic Respiratory Failure, Fungating Lung Mass, Interstitial Lung Disease, Lung Disease, Lung Nodule, Obesity Hypoventilation Syndrome, Obstructive Sleep Apnea, Oxygen Dependence, Pulmonary Contusion, Pulmonary Hypertension, Recurrent Aspiration, Recurrent Aspiration Pneumonia, Recurrent Pulmonary Nodular Amyloidosis, Restrictive Lung Disease, Sleep Apnea, Tuberculosis, Latent Tuberculosis |
| CKD | Chronic Kidney Disease, End Stage Renal Disease, Renal Insufficiency |
