# Supplementary material for: Predicting COVID-19 prognosis in hospitalized patients based on early status
Source: mBio. 2023 Sep 8;14(5):e01508-23. doi: 10.1128/mbio.01508-23 (PMC10653946; doi:10.1128/mbio.01508-23)
Supplement: Table S3 — Binning methods for hospital discharge. [file mbio.01508-23-s0007.docx]

**Supplemental Table 3. Binning Methods for Hospital Discharge**

| **Category** | **Discharge Types Included** |
| --- | --- |
| Acute Care | Field Hospital/Short Term Hospital |
| Chronic Care | Skilled Nursing Facility/Long-Term Acute Care Hospital/Long Term Care/Acute Care Facility/Facility/State Prison/Psychology Unit |
| Expired/Hospice | Death/Hospice/Home with Hospice/Nursing Home with Hospice |
| Home/Left Against Medical Advice | Home/Home with Rehab/Home Health Service/Assisted Living/Left Against Medical Advice |
