# Supplementary material for: Predicting COVID-19 prognosis in hospitalized patients based on early status
Source: mBio. 2023 Sep 8;14(5):e01508-23. doi: 10.1128/mbio.01508-23 (PMC10653946; doi:10.1128/mbio.01508-23)
Supplement: Table S4 — Comparison of CURB-65 performance at six cutoff values. [file mbio.01508-23-s0008.docx]

**Supplemental Table 4. Comparison of CURB-65 performance at six cutoff values**

| **Algorithm** | **Accuracy** | **Sensitivity** | **Specificity** | **PPV** | **NPV** | **F1** | **ROC AUC** | **Negative Log Loss** |
| --- | --- | --- | --- | --- | --- | --- | --- | --- |
| **CURB-65, cutoff 0** | 0.234 | 1.000 | 0.000 | 0.234 | 0.000 | 0.380 | 0.500 | -0.601 |
| **CURB-65, cutoff 1** | 0.526 | 0.930 | 0.403 | 0.323 | 0.949 | 0.479 | 0.666 | -0.601 |
| **CURB-65, cutoff 2** | 0.702 | 0.749 | 0.687 | 0.423 | 0.899 | 0.541 | 0.718 | -0.601 |
| **CURB-65, cutoff 3** | 0.752 | 0.335 | 0.880 | 0.461 | 0.812 | 0.388 | 0.607 | -0.601 |
| **CURB-65, cutoff 4** | 0.765 | 0.079 | 0.974 | 0.486 | 0.776 | 0.136 | 0.527 | -0.601 |
| **CURB-65, cutoff 5** | 0.768 | 0.013 | 0.999 | 0.75 | 0.768 | 0.026 | 0.506 | -0.601 |

Abbreviations: PPV, positive predictive value; NPV, negative predictive value; ROC AUC, Receiver Operating Characteristic Area Under Curve.
