# Supplementary material for: Predicting COVID-19 prognosis in hospitalized patients based on early status
Source: mBio. 2023 Sep 8;14(5):e01508-23. doi: 10.1128/mbio.01508-23 (PMC10653946; doi:10.1128/mbio.01508-23)
Supplement: Table S5 — Variables used in full model for mortality. [file mbio.01508-23-s0009.docx]

**Supplemental Table 5. Variables used in full model for mortality^a^**

| **Variable** | | **Patients who survived** | **Patients who died** | **P-Value** | **SMD^b,c^** |
| --- | --- | --- | --- | --- | --- |
|  | | 704 | 217 |  |  |
| Male, n (%) | | 428 (60.8) | 127 (58.5) | 1.000 | 0.046 |
| Age, median [Q1,Q3] | | 60.0 [48.0,71.0] | 74.0 [64.0,84.0] | <0.001 | 0.846 |
| Ethnicity, n (%) | African American | 101 (14.3) | 25 (11.5) | 0.007 | 0.403 |
|  | East Asian | 49 (7.0) | 15 (6.9) |  |  |
|  | Hispanic | 253 (35.9) | 47 (21.7) |  |  |
|  | Other Ethnicity | 26 (3.7) | 9 (4.1) |  |  |
|  | South Asian | 41 (5.8) | 10 (4.6) |  |  |
|  | White | 234 (33.2) | 111 (51.2) |  |  |
| Use of Interpreter, n (%) | | 186 (26.4) | 47 (21.7) | 1.000 | 0.112 |
| High Risk Contact, n (%) | | 167 (23.7) | 48 (22.1) | 1.000 | 0.038 |
| Smoking or Vaping, n (%) | | 91 (12.9) | 43 (19.8) | 1.000 | 0.187 |
| H/O Arrhythmia, n (%) | | 77 (10.9) | 48 (22.1) | 0.003 | 0.305 |
| H/O Coronary Artery Disease or MI, n (%) | | 118 (16.8) | 65 (30.0) | 0.002 | 0.316 |
| H/O DVT/PE, n (%) | | 30 (4.3) | 18 (8.3) | 1.000 | 0.167 |
| H/O Heart Failure, n (%) | | 68 (9.7) | 39 (18.0) | 0.097 | 0.243 |
| H/O Hypertension, n (%) | | 422 (59.9) | 156 (71.9) | 0.146 | 0.254 |
| H/O Cerebrovascular Disease, n (%) | | 60 (8.5) | 42 (19.4) | 0.001 | 0.317 |
| H/O Genitourinary Disease, n (%) | | 60 (8.5) | 33 (15.2) | 0.483 | 0.208 |
| H/O GI Disease, n (%) | | 123 (17.5) | 45 (20.7) | 1.000 | 0.083 |
| H/O Anemia, n (%) | | 49 (7.0) | 26 (12.0) | 1.000 | 0.172 |
| H/O Malignancy, n (%) | | 72 (10.2) | 48 (22.1) | 0.001 | 0.327 |
| H/O Diabetes, n (%) | | 273 (38.8) | 92 (42.4) | 1.000 | 0.074 |
| H/O Hyperlipidemia, n (%) | | 244 (34.7) | 105 (48.4) | 0.028 | 0.281 |
| H/O Hypothyroidism, n (%) | | 66 (9.4) | 26 (12.0) | 1.000 | 0.084 |
| H/O Musculoskeletal Disease, n (%) | | 92 (13.1) | 45 (20.7) | 0.582 | 0.206 |
| H/O Dementia, n (%) | | 75 (10.7) | 50 (23.0) | <0.001 | 0.336 |
| H/O Other Neurological Disease, n (%) | | 40 (5.7) | 15 (6.9) | 1.000 | 0.051 |
| H/O Anxiety, n (%) | | 38 (5.4) | 19 (8.8) | 1.000 | 0.131 |
| H/O Depression, n (%) | | 61 (8.7) | 28 (12.9) | 1.000 | 0.137 |
| H/O Asthma, n (%) | | 64 (9.1) | 10 (4.6) | 1.000 | 0.178 |
| H/O COPD, n (%) | | 49 (7.0) | 28 (12.9) | 0.659 | 0.200 |
| H/O Other Pulmonary Disease, n (%) | | 41 (5.8) | 20 (9.2) | 1.000 | 0.129 |
| H/O CKD, n (%) | | 64 (9.1) | 37 (17.1) | 0.121 | 0.238 |
| BMI, median [Q1,Q3] | | 27.9 [24.3,31.9] | 27.0 [23.5,31.6] | 1.000 | -0.089 |
| Temperature, median [Q1,Q3] | | 99.0 [98.2,100.7] | 99.0 [98.0,100.3] | 1.000 | -0.139 |
| Heart Rate, median [Q1,Q3] | | 99.0 [85.8,112.0] | 96.0 [84.0,110.0] | 1.000 | -0.036 |
| Respiratory Rate, median [Q1,Q3] | | 18.0 [17.8,22.0] | 18.0 [18.0,24.0] | 1.000 | 0.197 |
| Systolic Blood Pressure, median [Q1,Q3] | | 132.0 [118.0,146.0] | 123.0 [108.0,140.0] | 0.001 | -0.342 |
| Diastolic Blood Pressure, median [Q1,Q3] | | 75.0 [66.0,83.0] | 70.0 [60.0,78.0] | <0.001 | -0.403 |
| Oxygen Saturation, median [Q1,Q3] | | 94.0 [91.0,97.0] | 94.0 [89.0,97.0] | 1.000 | -0.209 |
| Patient Responsive, n (%) | | 657 (93.3) | 175 (80.6) | <0.001 | 0.384 |
| Altered Mental Status, n (%) | | 64 (9.1) | 52 (24.0) | <0.001 | 0.409 |
| Dry Mucous Membranes, n (%) | | 30 (4.3) | 22 (10.1) | 0.142 | 0.229 |
| Rales, n (%) | | 85 (12.1) | 36 (16.6) | 1.000 | 0.129 |
| Rhonchi, n (%) | | 48 (6.8) | 21 (9.7) | 1.000 | 0.104 |
| Tachypnea, n (%) | | 75 (10.7) | 20 (9.2) | 1.000 | 0.048 |
| Breath Sounds Equal, Both Sides Diminished, n (%) | | 38 (5.4) | 17 (7.8) | 1.000 | 0.098 |
| Duration of Symptoms More Than a Week, n (%) | | 304 (43.3) | 99 (45.6) | 1.000 | 0.047 |
| ALT, median [Q1,Q3] | | 34.0 [21.0,55.0] | 33.0 [20.0,52.0] | 1.000 | 0.048 |
| AST, median [Q1,Q3] | | 45.0 [32.0,71.0] | 56.0 [38.0,86.0] | 0.004 | 0.283 |
| Albumin, mean (SD) | | 3.6 (0.5) | 3.4 (0.5) | <0.001 | -0.433 |
| Alkaline Phosphatase, median [Q1,Q3] | | 75.0 [59.0,101.0] | 75.0 [60.0,104.5] | 1.000 | 0.081 |
| BUN, median [Q1,Q3] | | 15.0 [10.0,25.0] | 30.5 [19.0,49.8] | <0.001 | 0.615 |
| Bilirubin Total, median [Q1,Q3] | | 0.4 [0.3,0.6] | 0.5 [0.3,0.7] | 1.000 | 0.156 |
| C-Reactive Protein, median [Q1,Q3] | | 9.5 [4.8,16.4] | 11.8 [6.6,19.1] | 0.430 | 0.185 |
| Calcium, median [Q1,Q3] | | 8.7 [8.4,9.1] | 8.7 [8.2,9.1] | 1.000 | -0.107 |
| CO2, Total, median [Q1,Q3] | | 22.4 [20.3,24.2] | 21.1 [19.1,23.6] | 0.013 | -0.257 |
| Creatinine, median [Q1,Q3] | | 0.9 [0.7,1.2] | 1.3 [0.9,1.9] | <0.001 | 0.219 |
| D-Dimer Level, median [Q1,Q3] | | 1003.0 [560.5,1758.8] | 1272.0 [849.0,3962.0] | <0.001 | 0.327 |
| Eosinophil Count, Absolute, median [Q1,Q3] | | 0.0 [0.0,0.0] | 0.0 [0.0,0.0] | 0.056 | -0.180 |
| Ferritin, median [Q1,Q3] | | 698.5 [373.2,1344.8] | 861.0 [393.0,1591.0] | 1.000 | 0.179 |
| Fibrinogen, median [Q1,Q3] | | 694.0 [526.0,885.0] | 609.0 [455.5,837.0] | 1.000 | -0.256 |
| Glucose, median [Q1,Q3] | | 124.0 [108.0,162.0] | 139.5 [113.0,188.0] | 0.131 | 0.164 |
| Hemoglobin, median [Q1,Q3] | | 13.3 [12.0,14.4] | 12.8 [11.2,14.3] | 1.000 | -0.144 |
| INR, median [Q1,Q3] | | 1.2 [1.1,1.3] | 1.2 [1.1,1.3] | 0.662 | 0.100 |
| Lactate, median [Q1,Q3] | | 1.5 [1.1,2.0] | 2.1 [1.5,3.0] | <0.001 | 0.573 |
| LDH, median [Q1,Q3] | | 365.0 [269.0,489.0] | 401.0 [309.0,580.0] | 0.065 | 0.334 |
| Lymphocyte Count, Absolute, median [Q1,Q3] | | 0.9 [0.6,1.3] | 0.7 [0.5,1.1] | 0.006 | -0.145 |
| Magnesium, median [Q1,Q3] | | 2.1 [1.9,2.3] | 2.1 [1.9,2.5] | 1.000 | 0.221 |
| Monocyte Count, Absolute, median [Q1,Q3] | | 0.5 [0.4,0.7] | 0.5 [0.3,0.7] | 1.000 | 0.003 |
| MPV, median [Q1,Q3] | | 8.4 [7.9,9.1] | 9.0 [8.1,9.7] | <0.001 | 0.416 |
| Neutrophil Count, Absolute, median [Q1,Q3] | | 5.9 [4.2,8.1] | 6.8 [4.6,9.5] | 0.590 | 0.236 |
| Platelet Count, median [Q1,Q3] | | 229.5 [176.0,303.5] | 199.0 [154.0,275.0] | 0.007 | -0.310 |
| Potassium, median [Q1,Q3] | | 4.0 [3.7,4.4] | 4.2 [3.8,4.7] | 0.047 | 0.253 |
| RBC MCHC, median [Q1,Q3] | | 33.5 [32.7,34.1] | 33.0 [32.1,33.7] | <0.001 | -0.422 |
| RBC MCV, median [Q1,Q3] | | 87.4 [83.9,91.1] | 88.5 [84.8,93.2] | 0.189 | 0.247 |
| RDW, median [Q1,Q3] | | 14.1 [13.4,15.3] | 15.2 [14.0,17.1] | <0.001 | 0.460 |
| Serum Protein, Total, median [Q1,Q3] | | 7.3 [6.8,7.7] | 7.1 [6.6,7.5] | 0.038 | -0.261 |
| Sodium, median [Q1,Q3] | | 136.0 [133.0,139.0] | 137.0 [133.0,141.8] | 1.000 | 0.232 |
| Troponin T, Elevated, n (%) | | 79 (15.5) | 64 (38.8) | <0.001 | 0.542 |
| WBC, median [Q1,Q3] | | 7.7 [5.7,10.2] | 8.6 [6.2,11.8] | 0.132 | 0.271 |

^a^Bonferroni-corrected Kruskal-Wallis H test was used for all continuous variables. Chi squared tests were used for all categorical variables.

^b^Abbreviations: SMD, standardized mean difference; Q1, first quartile; Q3, third quartile; SD, standard deviation; H/O, history of; MI, myocardial infarction; DVT/PE, deep vein thrombosis/pulmonary embolism; GI, gastrointestinal; COPD, chronic obstructive pulmonary disease; CKD, chronic kidney disease; BMI, body mass index; ALT, alanine aminotransferase; AST, aspartate aminotransferase; BUN, blood urea nitrogen; INR, international normalized ratio; LDH, lactate dehydrogenase; MPV, mean platelet volume; RBC, red blood cell; MCHC, mean corpuscular hemoglobin concentration; MCV, mean corpuscular volume; RDW, red cell distribution width; WBC, white blood cell.

^c^Standardized mean difference (SMD) was used for assessment of effect size, with positive values indicating direct relationships, and negative values inverse relationships.
